# Simulation Reveals the Chameleonic Behavior of Macrocycles

**DOI:** 10.1021/acs.jcim.2c01093

**Published:** 2022-12-23

**Authors:** Daniel Sethio, Vasanthanathan Poongavanam, Ruisheng Xiong, Mohit Tyagi, Duc Duy Vo, Roland Lindh, Jan Kihlberg

**Affiliations:** Department of Chemistry − BMC, Uppsala University, Box 576, SE-751 23Uppsala, Sweden

## Abstract

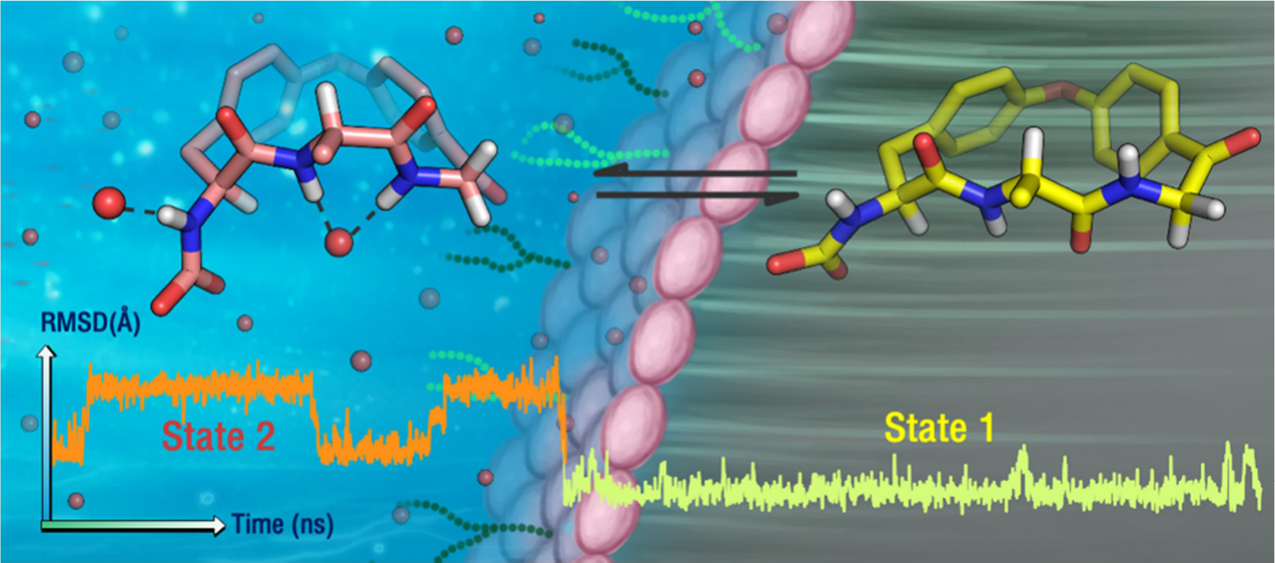

Conformational analysis is central to the design of bioactive molecules. It is particularly challenging for macrocycles due to noncovalent transannular interactions, steric interactions, and ring strain that are often coupled. Herein, we simulated the conformations of five macrocycles designed to express a progression of increasing complexity in environment-dependent intramolecular interactions and verified the results against NMR measurements in chloroform and dimethyl sulfoxide. Molecular dynamics using an explicit solvent model, but not the Monte Carlo method with implicit solvation, handled both solvents correctly. Refinement of conformations at the *ab initio* level was fundamental to reproducing the experimental observations—standard state-of-the-art molecular mechanics force fields were insufficient. Our simulations correctly predicted the intramolecular interactions between side chains and the macrocycle and revealed an unprecedented solvent-induced conformational switch of the macrocyclic ring. Our results provide a platform for the rational, prospective design of molecular chameleons that adapt to the properties of the environment.

## Introduction

Macrocycles are prominent among natural products and synthetic bioactive molecules, with vital roles in fields such as supramolecular chemistry,^1^ artificial molecular machines,^2^ and drug discovery.^3−5^ Their unique properties originate from the molecular-level preorganization of structural elements induced by macrocyclization. Predicting the conformational landscape of molecules is essential as it determines their physical and biomolecular properties, but conformational analysis of macrocycles is complex and challenging.^6,7^ Reasons for this include that macrocycles often have a large number of degrees of freedom, form noncovalent transannular interactions such as intramolecular hydrogen bonds (IMHBs), and that steric interactions and ring strain also influence macrocycle conformations. Large and flexible side chains further increase the complexity of macrocycle conformational analysis, in particular, as the flexibility of the ring may amplify the overall flexibility.^8,9^ Nonbonded intramolecular interactions between side chains and the macrocyclic ring allow macrocycles to behave as molecular chameleons^8−13^ a feature which may provide major advantages in drug discovery.^12^ However, the design of macrocyclic molecular chameleons remains an important yet unresolved problem.

In general, the conformational space populated by macrocycles is sampled well by different algorithms,^14,15^ but the identification of the biologically relevant conformations, i.e., solution- and/or target-bound conformations, remains a formidable challenge.^16,17^ Recently, progress has been made for macrocyclic peptides which may form transannular IMHBs.^18−20^ However, for nonpeptidic and semipeptidic macrocycles, minimum energy conformations (MECs) identified by molecular mechanics using implicit solvation models show large differences from the biologically relevant conformations, while conformations resembling the biologically relevant ones usually reside at ≥10–15 kcal mol^–1^ above the MECs.^16,17,21^ Hence, this calls for the development of computational protocols which correctly model the behavior of macrocycles in a variety of environments such that the predicted properties can guide the design of molecules for specific purposes. The need for the prospective, property-based design of macrocycles^3,22^ is further underlined by the complexity of their synthesis.^23^

**Figure 1:**
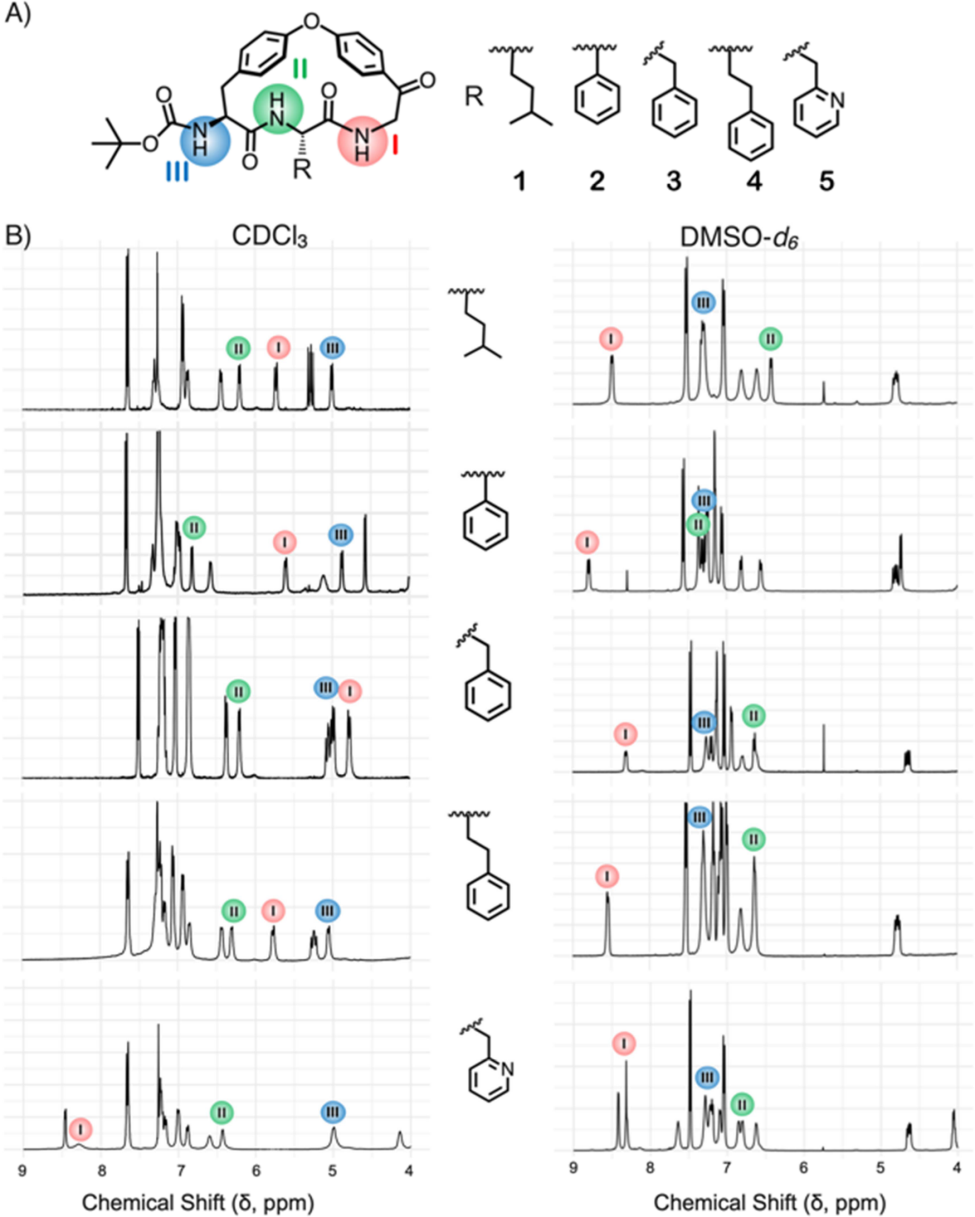
(A) Structures of the macrocycles investigated in this study. (B) ^1^H NMR spectra of **1**–**5** in CDCl_3_ and DMSO-d_6_. Chemical shifts of each of the three amide protons NH-I (red), NH-II (green), and NH-III (blue) are indicated.

Inspired by the natural products hymenocardine,^24^ K-13,^25^ and OF4949-III,^26^ we designed macrocycles **1**–**5** that contain an 18-membered macrocyclic ring with two side-chains (R and Boc, Figure 1A).^27^ The R side-chain was chosen to allow the formation of an intramolecular NH···π interaction (**2**–**4**) or a hydrogen bond (**5**) with either of the adjacent amide bonds. The chemical shifts of the three amide protons of **1**–**5** indicate major structure- and environment-dependent conformational differences between **1**–**5** (Figure 1B, Supplementary Figures S1–S12, Tables S1 and S2). For instance, the chemical shift of NH-I shows a large variation between **1**–**5** in a nonpolar environment (CDCl_3,_ ε = 4.8) but not in the polar one (DMSO-*d*_6_, ε = 46.7). As chemical shifts are sensitive to molecular structure and dynamics,^28−32^ macrocycles **1**–**5** constitute an ideal system for the development and assessment of computational protocols to predict macrocycle conformations. Herein, we report our successful efforts to this end. Molecular dynamics (MD) simulations, followed by DFT optimization of the generated conformational ensembles, allowed the correct prediction of solvent-dependent dynamic intramolecular interactions and of a conformational switch of the ring in the series of macrocycles.

## Methods

### Monte Carlo Conformational Sampling (MCMM)

The 2D structures of the designed macrocycles **1**–**5** were built with correct stereochemistry using Maestro from the Schrödinger suite.^33^ The resulting initial structures were directly used as input for the Monte Carlo Multiple Minimum (MCMM) Conformational Sampling using implicit solvation models.

The MCMM conformational samplings were conducted using the Macromodel software as implemented in the Schrödinger Suite version 2020-4.^34^ The MCMM sampling^35^ was done in both polar and nonpolar environments as represented by dimethyl sulfoxide (DMSO, ε = 46.7) and chloroform (CHCl_3_, ε = 4.8) using the OPLS3e force field.^36^ Briefly, the conformational space of each macrocycle in each of the two solvents was stochastically explored by extended sampling of the torsional angles. The following settings were used for the study: energy window (10 kcal/mol), elimination of duplicate conformer threshold (RMSD, 0.75 Å), the total number of iterations (10,000 steps), and force field [Polak-Ribière Conjugate Gradient (PRCG) in combination with the OPLS3e force field^36^]. For each kept unique conformation (within the 10 kcal/mol window, RMSD >0.75 Å relative to conformations identified earlier in the sampling), a degeneracy index was assigned, indicating how many times identical conformations were found in the iterative sampling. Finally, the 10 conformations that had the highest degeneracy index were selected. The **MC** protocol used the MEC from the MCMM calculations for each compound in each of the two solvents to calculate the chemical shifts of NH-I, NH-II, and NH-III. Subsequently, all conformations (10 per compound and solvent) were subjected to DFT-based geometry optimization as described below.

### MD Simulations

All MD simulations for compounds **1**–**5** were performed using the Amber18 package^37^ utilizing the General Amber Force Field (GAFF).^38^ The initial geometry for **1**–**5** was taken from the lowest energy conformer as obtained from the MCMM calculation. Charges for atoms were assigned based on the electrostatic potential (ESP) fitting using the RESP procedure^39^ as implemented in Antechamber software.^40^ The ESP calculation was carried out at the B3LYP/cc-pVTZ level of theory, using the Gaussian 16 Rev. C.01 software.^41^ Solvent molecules (CHCl_3_ or DMSO) were added to the macrocycles with 30 Å buffering distance between the edges of the truncated cubic box. Subsequently, the *tleap* tool^42^ from the Amber package was used to build topology and coordinate files for carrying out the MD calculations. Initially, energy minimization was carried out in two steps. First, the steepest descent method was used to minimize the energy with respect to the hydrogen atom positions with a maximum of 1000 steps, whereas heavy atoms were constrained. Second, the conjugate gradient method with 2000 steps was used to minimize the whole solute–solvent system. Subsequently, the system was heated from 0 to 300 K at a constant volume for 200 ps. After the thermalization process, the system was equilibrated at constant temperature (300 K) and pressure (1 bar) using the Berendsen coupling algorithm^43^ for another 500 ps MD simulation. Finally, the MD production run was performed for 50 ns for each compound in the two solvents. The SHAKE algorithm^44^ was used to constrain all bonds involving hydrogen atoms. Snapshots were collected every 10 ps from the 50 ns simulation, resulting in a total of 5000 snapshots for further analysis for each compound and solvent. A nonbiased selection of 10 of the 5000 snapshots provides a representative subset with minimal likelihood of not including the major conformation and several high-energy conformations. Every 500th conformation was therefore chosen, and the energy was calculated at the B3LYP/6-311++G(2d,p) level of theory. The **MD** protocol used the MEC out of these 10 conformations for each compound in each of the two solvents to calculate the chemical shifts of NH-I, NH-II, and NH-III. Subsequently, all conformations (10 per compound and solvent) were subjected to DFT-based geometry optimization as described below.

### Trajectory Analysis

The conformational change of macrocycles was followed through RMSD, inter- and intramolecular hydrogen bonding, and NH···π interactions utilizing the *cpptraj* tool.^45^ The RMSD values were calculated with respect to the initial geometry, while the NH···π interactions were evaluated by measuring the distance between the N atom to the center of the phenyl ring. The flexibility of the dipeptide of the macrocyclic ring was monitored through the change of the two dihedral angles ϕ and ψ (see the Supporting Information).

### Geometry Optimization (DFT)

The 10 conformations obtained from the MCMM calculations and the MD simulations for each compound in each of the two solvents were further optimized at the DFT level using the Gaussian 16 Rev. C.01 program.^41^ To find a reliable and practical method for describing nonbonded interactions, several DFT functionals and basis sets have been tested. The dispersion-corrected functionals such as B3LYP^46,47^ augmented with the semiempirical Grimme’s D3 dispersion corrected,^48^ M06-2X,^49^ and wB97X-D^50^ functionals have been tested together with Pople’s 6-31+G(d,p)^51^ and 6–31++G(d,p)^52^ as well as Dunning’s cc-pVDZ and cc-pVTZ basis sets.^53,54^ The M06-2X functional in combination with Pople’s 6-31 + G(d,p) was chosen as the best compromise to accurately describe nonbonded interactions as suggested by Willoughby and co-workers.^55^ The implicit polarizable continuum model of Tomasi and co-workers was used to account for CHCl_3_ and DMSO solvation effects.^56^ To ensure the optimized geometry corresponding to a minimum on the potential energy surface, vibrational frequency calculations were conducted at the same level of theory. The MECs for each compound in each of the two solvents were used to calculate the chemical shifts of NH-I, NH-II, and NH-III in the **MC:DFT** and **MD:DFT** protocols. All 10 conformations were used for the **MC:DFT_ens_** and **MD:DFT_ens_** protocols.

### NMR Chemical Shift Calculations

NMR chemical shielding(s) calculations were calculated at the B3LYP/6-311++G(2d,p) level of theory utilizing the Gauge-Independent Atomic Orbital (GIAO) method.^57^ The triple-ξ polarized quality augmented with the diffuse functions basis set is necessary to obtain accurate NMR shielding tensor values.^58^ The NMR chemical shifts are obtained by subtracting the chemical shielding tensor value of the reference compound with the chemical shielding tensor value of the molecule of interest (δ = σ_ref_ – σ). Tetramethyl silane (TMS) was used as a reference compound for calculating the GIAO tensor of ^1^H NMR.

### Model Evaluation

Prediction of relevant conformations was evaluated in the following scenario, if the minimum energy conformer (MEC) is representative (or not) of the conformations populated by the compound in different environments.

### Interproton Distances (NOEs)

To obtain quantitative inter-proton distances, seven ^1^H,^1^H-NOESY experiments were evaluated with increasing mixing times ranging from 50 to 350 ms. Experiments were recorded in random order to counteract systematic errors. Spectra were acquired with 1024 × 2048 complex points (F1 × F2) and a spectral width of 6410 Hz. The recycle relaxation delay (*d*1) was set to 3 s. Deuterium signals of CDCl_3_ and DMSO-*d*_6_ were used for the lock signal. Diagonal and cross peaks were integrated for all mixing times and individual NOE intensities taken as the absolute integral were normalized according to
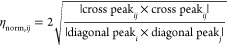
1per mixing time, respectively. A minimum of four normalized intensities, η_norm_, for consecutive mixing times describing a linear initial build-up with *R*^2^ ≥ 0.95 were used to determine the build-up rate σ_*ij*_. Build-up rates of locked-in-distance protons were used as distance reference (e.g., geminal protons: 1.78 Å). Distances were calculated according to
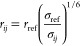
2where *r_ij_* is the resulting distance between protons *i* and *j*, *r*_ref_ is the fixed distance of the reference proton pair, σ_ref_ is the build-up rate of the used reference protons, and σ_*ij*_ is the build-up rate of the protons of interest using normalized NOE intensities.^59^

## Results and Discussion

### Study Design

First, protocols to identify the conformational dynamics of macrocycles as a function of the polarity of the environment, i.e., apolar (chloroform) versus polar (DMSO) were developed. Second, the robustness of the protocols was evaluated by a comparison between theoretical and experimental results, i.e., the chemical shifts of protons NH-I, NH-II, and NH-III (Figure 1),^27^^3^*J*_H,H_ scalar coupling constants, and nuclear Overhauser effects (NOEs) of protons within the macrocyclic ring of **1**–**5**. For the first part, six different protocols were explored to generate representative conformations of **1**–**5** (Table 1). Initial conformational sampling was performed either by the MCMM method, which incorporated an implicit solvation model, or by the more computationally intensive unrestrained-MD simulations using an explicit solvation model. These sampling methods are based on state-of-the-art force fields and are denoted **MC** and **MD**, respectively, when their MECs are used as representative conformations. An improved level of conformational sampling was established by *ab initio* DFT molecular geometry optimization of ten conformations selected from the established **MC** and **MD** ensembles (cf. Methods). We denote the use of the MECs from these optimized ensembles protocols **MC:DFT** and **MD:DFT**. Finally, the full ensembles consisting of ten conformations for each macrocycle constitute the **MC:DFT_ens_** and **MD:DFT_ens_** protocols, respectively. We note that the MECs of the **MC:DFT** protocol were used as a seed for subsequent MD conformational exploration.

**Table 1:** Protocols^a^ Evaluated for Conformational Sampling of Macrocycles **1–5** and the Quality of the Models for the Prediction of the Chemical Shifts of NH-I, NH-II, and NH-III^b^

protocol^a^	solvation model	CHCl_3_ (ε 4.8)		DMSO (ε 46.7)	
		*R*^2^	RMSE	*R*^2^	RMSE
**MC**	implicit	0.13	1.16	–0.32	2.72
**MC:DFT**	implicit	0.81	0.57	0.0003	2.41
**MC:DFT_ens_**	implicit	0.91	0.68	–0.002	2.17
**MD**	explicit	0.29	0.93	0.02	1.82
**MD:DFT**	explicit	0.88	0.66	0.63	1.26
**MD:DFT_ens_**	explicit	0.97	0.45	0.59	0.95

a MC: Monte Carlo Multiple Minimum; MD: Molecular Dynamics; DFT: Density Functional Theory. The subscript “ens” indicates that the model is based on the average of the chemical shifts of the conformational ensemble within 10 kcal mol^–1^ of the MEC.

b The quality of the protocols are described by the Pearson’s correlation coefficient (*R*^2^) and the root mean square error (RMSE, δ, ppm).

### Protocol for Finding Relevant Conformations of Macrocycles

**Figure 2:**
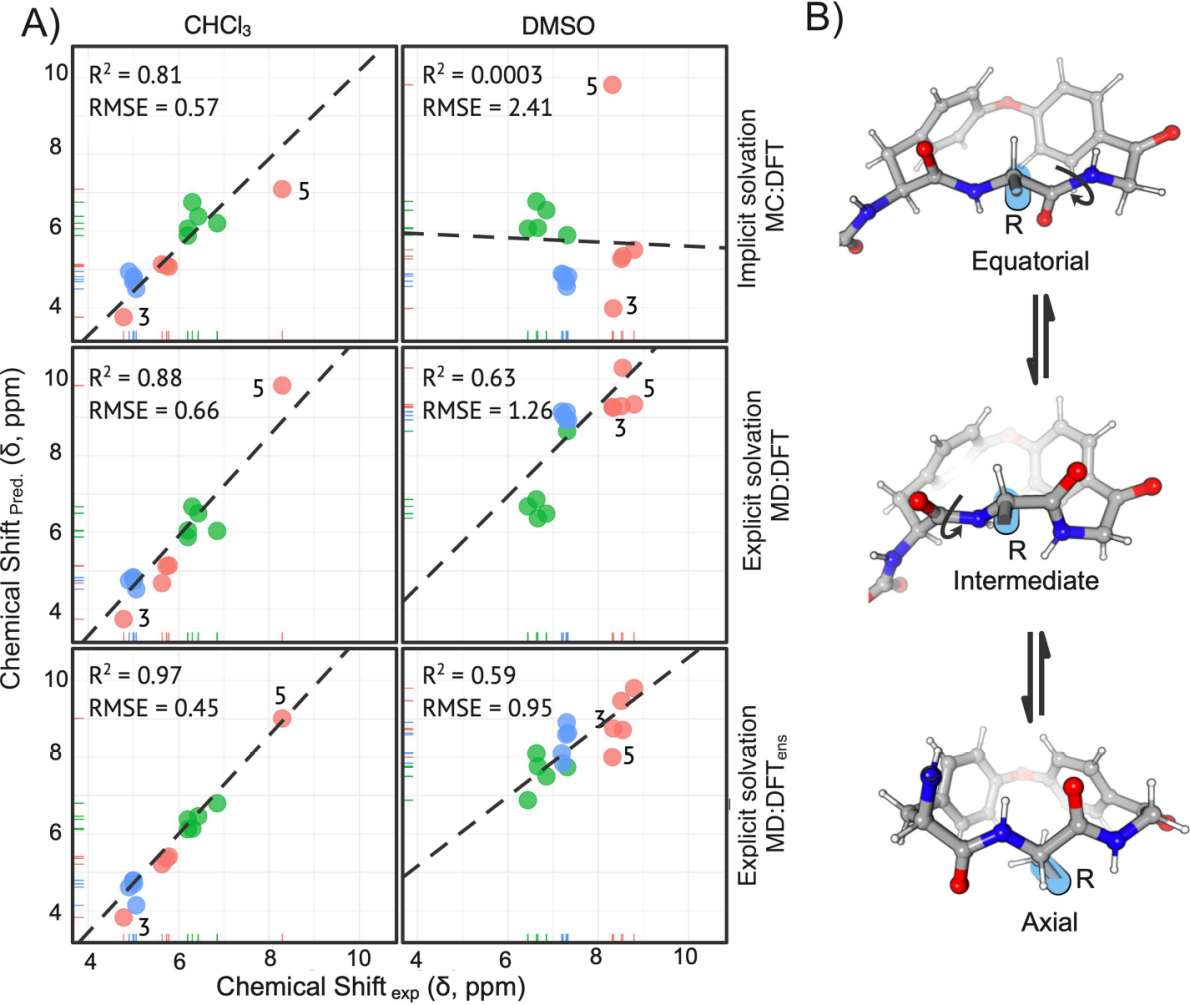
(A) Correlation between predicted and experimental ^1^H chemical shifts in chloroform and DMSO for compounds **1**–**5**. The top two panels show the correlations obtained for the MECs from protocol **MC:DFT**, the middle panels show the correlations obtained for the MECs from protocol **MD:DFT**, while the two panels at the bottom show the correlations based on the **MD:DFT_ens_** ensemble within 10 kcal mol^–1^ of the MEC. *R*^2^: the Pearson correlation coefficient; RMSE: root-mean-square-error (ppm). (B) Schematic description of the conformational interconversion of the macrocyclic ring that results in the reorientation of the R side-chain from and equatorial to an axial position.

As expected from previous reports,^16,17^ the MECs predicted by MC sampling were not representative of the solution conformations of **1**–**5** (Table 1, protocol **MC**, Supplementary Figure S13). The **MC:DFT** protocol provided a significant improvement in the correlation between predicted and observed chemical shifts for the MECs in chloroform, but not in DMSO (Table 1, Figure 2A). Inspection of the MECs from this protocol indicated that the shielding and deshielding of NH-I observed for compounds **3** and **5** in chloroform (Figure 1B) originated from the formation of an intramolecular NH···π interaction or hydrogen bond with the adjacent R phenyl and 2-pyridyl groups, respectively. The IMHB was maintained in implicit DMSO, providing one explanation for the poor correlation in this solvent. Averaging of chemical shifts calculated from the DFT-optimized conformational ensembles within an energy window of 5, 10, 15, and 20 kcal mol^–1^ did not provide any major improvement (Table 1, protocol **MC:DFTens**, Supplementary Figure S14). An implicit solvation model thus failed to reproduce solvation effects in the polar solvent DMSO, while it provided a good representation of the conformations adopted in an apolar environment.^60,61^ We, therefore, turned to unrestrained MD simulations in explicit solvents to capture the solvation effects on the conformations of **1**–**5**.

The MECs of protocol **MC:DFT**, all had the R side-chain oriented equatorially on the macrocyclic ring (Supplementary Figure S15), but the ensembles also contained conformations in which both amide bonds within the macrocycle had been rotated in the plane, thereby placing R axially (Figure 2B). In chloroform, conformations that had R axially had energies higher than that of the MEC (>3–22 kcal mol^–1^) (Supplementary Table S3), suggesting the equatorial conformations as starting points for the MD simulations. To understand whether these two minima are connected in the potential energy surfaces, we performed MD simulations at increasing temperatures for compound **5** in DMSO, since **5** may be particularly prone to remain in a local minimum due to the formation of an IMHB between the pyridyl group and NH-I. These simulations showed a transition from the axial to the equatorial orientation of the R side-chain at 500 K (Supplementary Figure S16), revealing conformations having R equatorial as suitable starting points for the MD simulations also in DMSO.

The MECs from the **MD** protocol provided poor correlations to the experimental chemical shifts both in chloroform and DMSO (Table 1, Supplementary Figure S17) probably due to an inadequate description of the solvation effects by the force field. However, the analysis of the frequency of hydrogen bonds revealed that IMHBs found in chloroform were broken up in DMSO and that the amide protons of **1**–**5** were hydrogen-bonded to DMSO (Supplementary Figures S18 and S19). DFT optimization, protocol **MD:DFT**, of the conformations, including the hydrogen-bonded DMSO molecules, led to a major improvement of the chemical shift correlations for the MECs both in chloroform and in DMSO (Table 1, Figure 2A). Lastly, the use of the <10 kcal mol^–1^ ensembles of protocol **MD:DFT_ens_** provided even better models in both solvents (Table 1, Figure 2A, Supplementary Figure S20) than the **MD:DFT** protocol. The ^3^*J*_H,H_ coupling constants for the protons in the macrocyclic ring of **1**–**5** were used to further validate the protocols. Encouragingly, the simulated coupling constants for the MECs of protocol **MD:DFT** agreed well with the experimentally determined ^3^*J*_H,H_ values both in chloroform and in DMSO (Supplementary Tables S4 and S5). This was also the case for the optimized MECs of the **MC:DFT** sampling protocol in chloroform, but not in DMSO.

### Validation of the Ability of the MD:DFT_ens_ Protocol To Predict Molecular Chameleons

**Figure 3:**
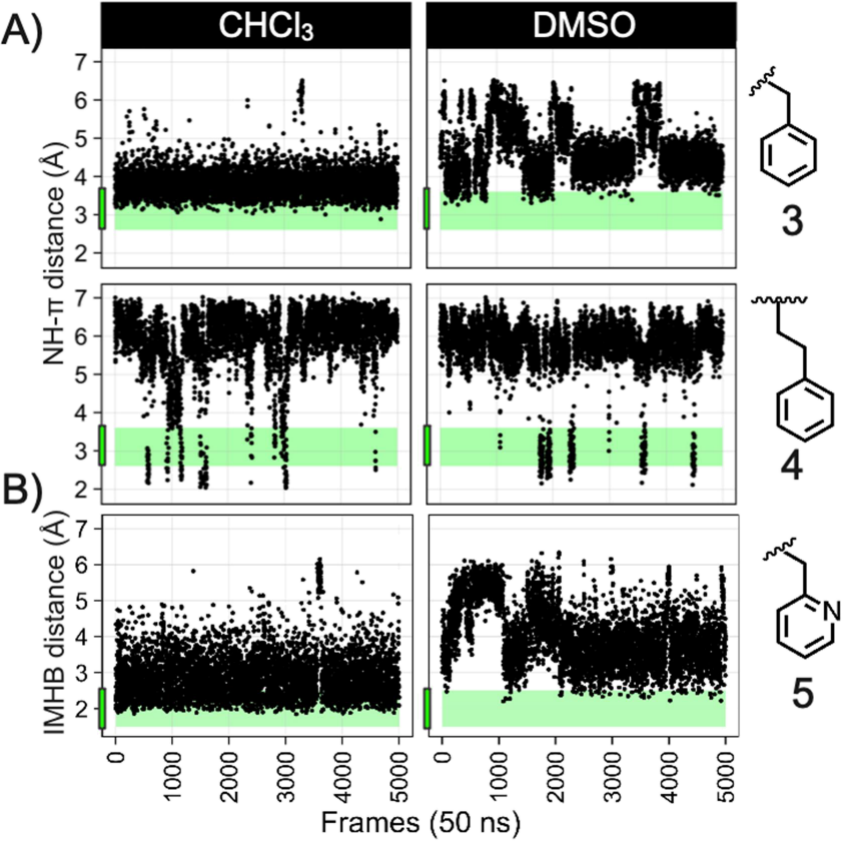
Distance between (A) the nitrogen atom of NH-I and the center of the R phenyl ring and (B) the nitrogen atom of NH-I and the nitrogen atom of the pyridyl moiety in the MD trajectories of compounds **3**, **4**, and **5**, respectively, in DMSO and CHCl_3_. Distance range for the formation of a NH···π shielding (2.9–3.6 Å) and non-negligible IMHB (1.5–2.5 Å) are highlighted in green.

The MD simulations suggested that the phenyl ring of phenylalanine **3** formed a significantly larger proportion of NH···π interactions with NH-I in chloroform than for homophenylalanine **4** (Figure 3A). Experimental support is provided by the large shielding of NH-I of **3** in chloroform as compared to **4** (Figure 1). For compound **5**, NH-I forms an IMHB to the pyridine moiety with high frequency in chloroform (Figure 3B), as expected from the large deshielding of NH-I observed in the NMR spectrum of **5** (Figure 1). These nonbonded interactions are disrupted in the MD simulations of **3** and **5** in explicit DMSO (Figure 3), in excellent agreement with that shielding/deshielding of NH-I are not observed in DMSO (Figure 1).

**Figure 4:**
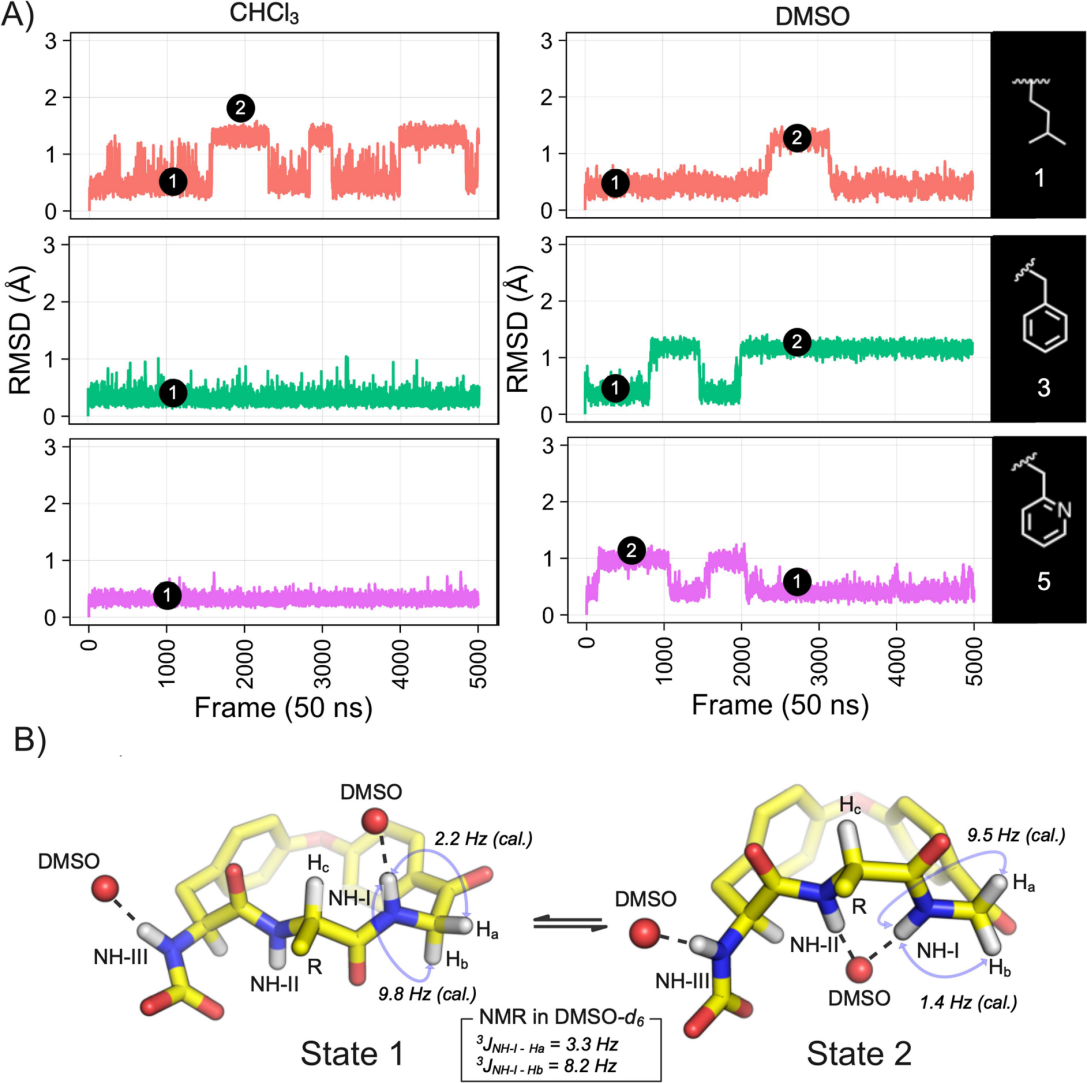
(A) RMSD of the heavy atoms of the macrocyclic ring of compounds **1**, **3**, and **5** calculated from the MD trajectories in explicit chloroform and DMSO. Representative conformations having the ring in states 1 and 2 have been indicated for each compound. (B) Structures of the state 1 and 2 conformations adopted in DMSO. The ^3^*J*_H,H_ coupling constants and NOEs for protons NH-I and H_a_–H_c_ provide key information that differentiates the two states. Coupling constants calculated for the state 1 and 2 conformations of the MEC from the **MD:DFT** protocol of compound **3** in DMSO have been included in the figure. Coupling constants determined by NMR spectroscopy in DMSO-*d*_6_ for **3** have been inserted in the box for comparison.

The MD trajectories of compounds **1**–**5** in the two environments also suggested that the macrocyclic ring populates a few major conformations (Supplementary Figure S21). For **1**, **3**, and **5**, the ring appeared to switch between two major conformations with **1** showing this behavior both in chloroform and DMSO, while **3** and **5** displayed it only in DMSO (Figure 4A). The conformations from protocol **MD:DFT_ens_** of **1**, **3**, and **5** revealed that the two conformational states originated from an in-plane rotation of the NH-I amide bond (Figure 4B). This places NH-I and NH-II on opposite faces of the macrocycle in state 1 and on the same face in state 2. The DFT-optimized MECs for the three macrocycles belong to state 1 both in chloroform and in DMSO. In chloroform, the second state of **1** was found to reside >20 kcal mol^–1^ above the MEC, i.e. the population of state 2 is negligible for **1** just as suggested by the MD simulations for **3** and **5** (Supplementary Table S6). However, in DMSO, the relative population of state 2 was indicated to be significant for all three macrocycles due to the coordination of a DMSO molecule by NH-I and NH-II (Figure 4).

The ^3^*J*_H,H_ coupling constants and NOEs for protons NH-I, H_a_, and H_b_ support the solvent-induced switch of the macrocyclic ring (Figure 4). In chloroform, the experimentally determined coupling constants between NH-I and the two adjacent methylene protons agree very well with the coupling constants predicted for state 1 (Supplementary Table S7), providing experimental support for the population of only this state in an apolar environment. In DMSO, the ^3^*J*_NH-I-Hb_ coupling constant determined by NMR spectroscopy lies in between the ones calculated for the state 1 and 2 conformations for the three macrocycles (exemplified for **3** in Figure 4B, Table S8). This is also the case for ^3^*J*_NH-I-Ha_ for **3**, whereas line-broadening prevented analysis for **1** and **5**. The coupling constants thus support that **1**, **3**, and **5** interconvert rapidly between the state 1 and 2 conformations in DMSO. NOESY confirmed the existence of the solvent-induced conformational switch. Specifically, NH-I displays an observable NOE only to one of the adjacent methylene protons (H_a_) in chloroform, but to both methylene protons in DMSO (Figure 4B, Supplementary Table S9, Figures S22–S27). In addition, the distance between NH-I and methine proton H_c_ was determined to be longer in DMSO than in chloroform for **1**, **3**, and **5** as would be expected from the population of both states in DMSO (Supplementary Table S9).

## Conclusions

Herein, we have reported the successful prediction of the conformational ensembles of a series of macrocycles in environments that differ in polarity and hydrogen bonding capacity. Monte Carlo conformational sampling in implicit solvent, followed by DFT optimization, provided an excellent description of the conformations populated in chloroform, but not so in the polar DMSO. The lack of predictivity of implicit solvent models in polar environments has been noted earlier for macrocycles^16,17,21^ and was comprehensively illustrated in a most recent study of linear peptides.^62^ However, we found that MD simulations using explicit solvation followed by DFT optimization described the conformations of the macrocycles correctly in both environments. These observations indicate that hydrogen bonding dictates the use of explicit solvent models for correct predictions in polar solvents.

Our MD simulations suggested that DMSO induced a conformational switch of the macrocyclic ring, while chloroform did not. Determination of coupling constants and NOEs by NMR spectroscopy verified this dynamic, solvent-dependent conformational flexibility. As the solvent-dependent formation of intramolecular NH···π and IMHB interactions between side-chains and the macrocyclic backbone were also correctly predicted, we conclude that MD simulations, followed by DFT optimization, can be used for the prospective design of molecular chameleons. Molecular chameleonicity is of major interest in drug discovery as it allows compounds to display high aqueous solubility and high cell permeability, properties that are highly desired but often difficult to engineer into a drug candidate.

## Supporting Information Available

Tables and figures with calculated data and NMR data; and experimental procedures for the synthesis of **5** (PDF)
Excel showing SMILES of compounds **1**−**5** (XLSX)
Excel showing chemical shifts statistics (XLSX)
RMSD analysis (XLSX)
Dihedral angle analysis (XLSX)
Distance and IMHB analysis (XLSX)

## Notes

MD trajectories and molecular topology parameter files from the MD simulations are available free of charge at https://doi.org/10.5281/zenodo.7022549. Python scripts used for automation of the calculation of chemical shifts and optimized geometries are available free of charge at https://github.com/danielquantum/NMR-Macrocycles.
