# Supplementary material for: Simulation Reveals the Chameleonic Behavior of Macrocycles
Source: J Chem Inf Model. 2022 Dec 23;63(1):138–46. doi: 10.1021/acs.jcim.2c01093 (PMC9832480; doi:10.1021/acs.jcim.2c01093)
Supplement: Supplementary file 1 — ci2c01093_si_001.pdf [file ci2c01093_si_001.pdf]

Supporting Information

Simulation Reveals the Chameleonic Behavior of Macrocycles

Daniel Sethio,[‡] Vasanthanathan Poongavanam,[‡] Ruisheng Xiong, Mohit Tyagi, Duc Duy Vo, Roland Lindh and Jan Kihlberg*

Department of Chemistry – BMC, Uppsala University, Box 576, SE-751 23 Uppsala, Sweden

*Corresponding author: jan.kihlberg@kemi.uu.se

Table of Contents

Supplementary data **2**

Synthesis of compound 5 .... **33**

General methods *33*

Synthetic scheme for compound 5 .... *34*

Experimental procedures *34*

NMR spectra of compounds S3, S5–5 .... *38*

References **47**

Supplementary data

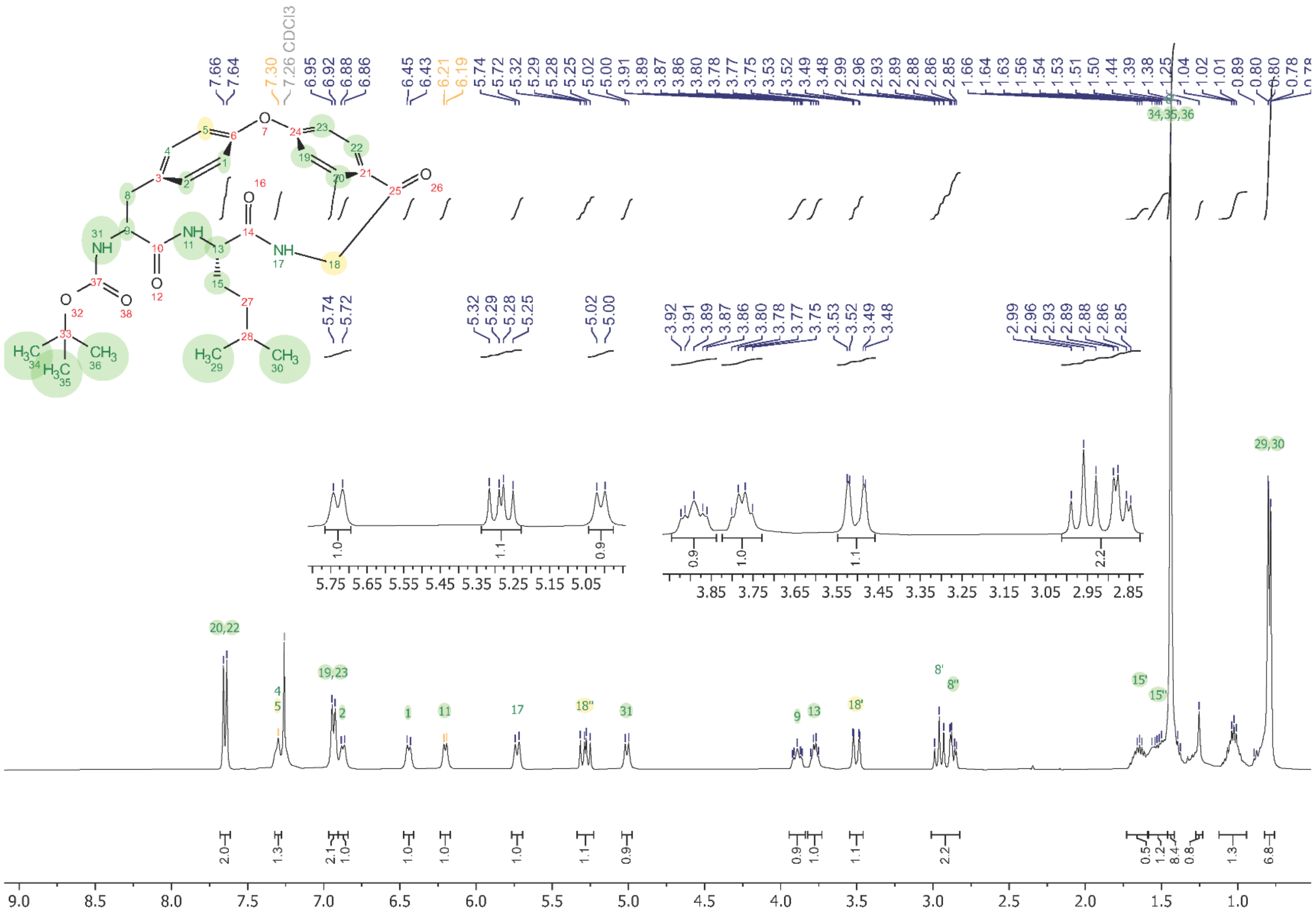

Figure S1. ^{1}H NMR spectrum of compound **1** in $CDCl_3$ recorded at 25 °C at 400 MHz.

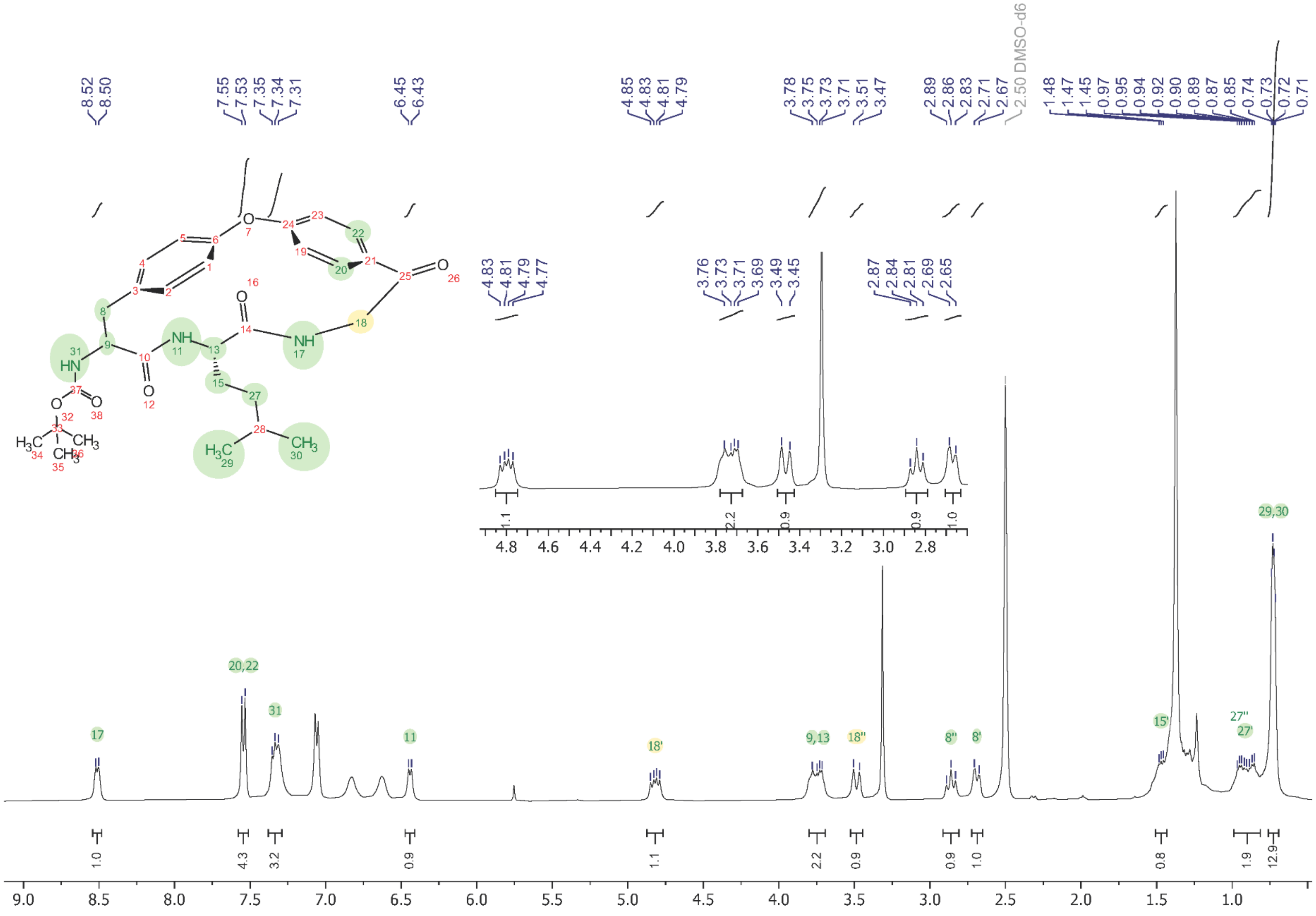

Figure S2. ^{1}H NMR spectrum of compound **1** in DMSO-d_6 recorded at 25 °C at 400 MHz.

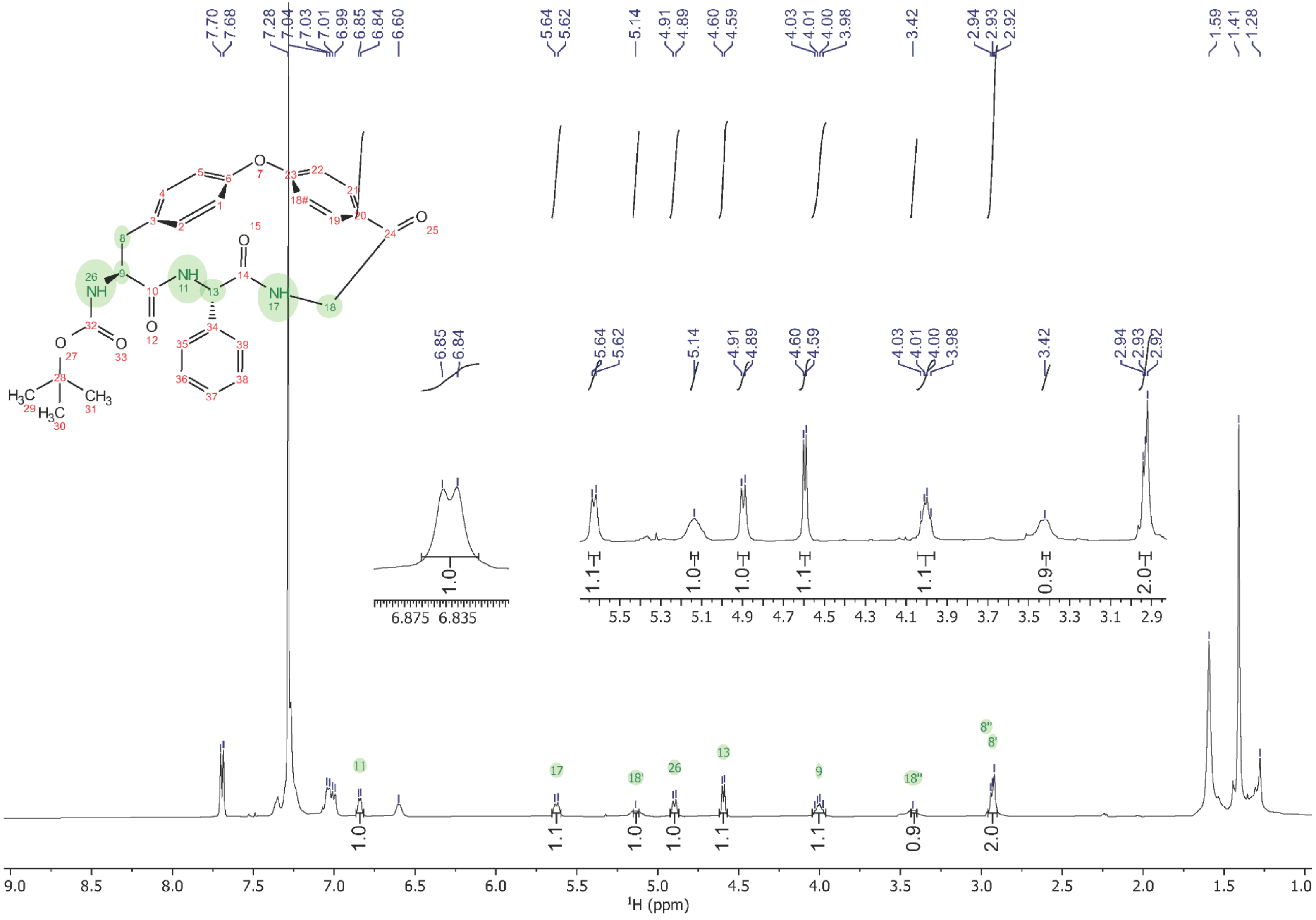

Figure S3. ^{1}H NMR spectrum of compound **2** in $CDCl_3$ recorded at 25 °C at 500 MHz..

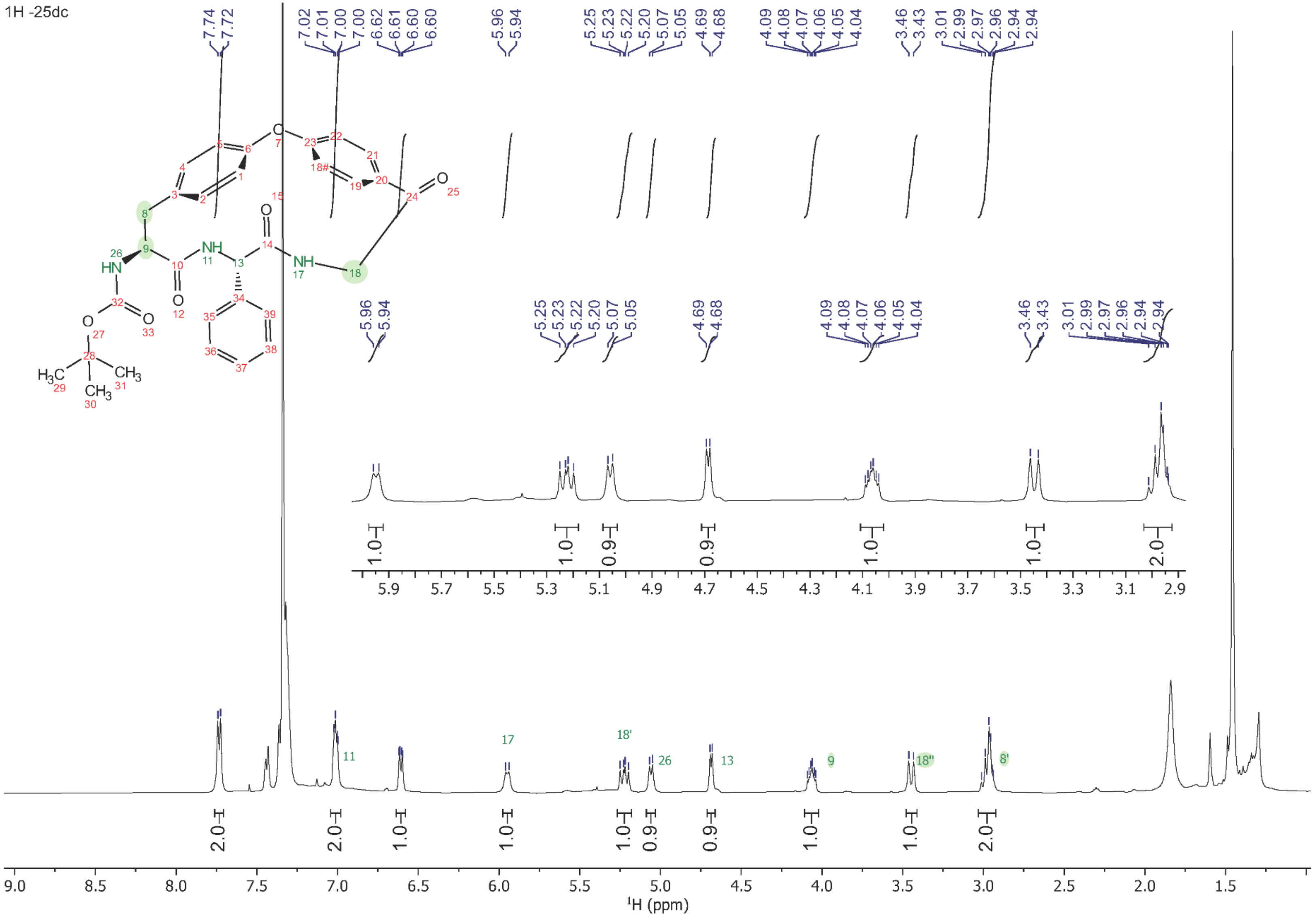

Figure S4. ^{1}H NMR spectrum of compound **2** in $CDCl_3$ recorded at -25 °C at 500 MHz..

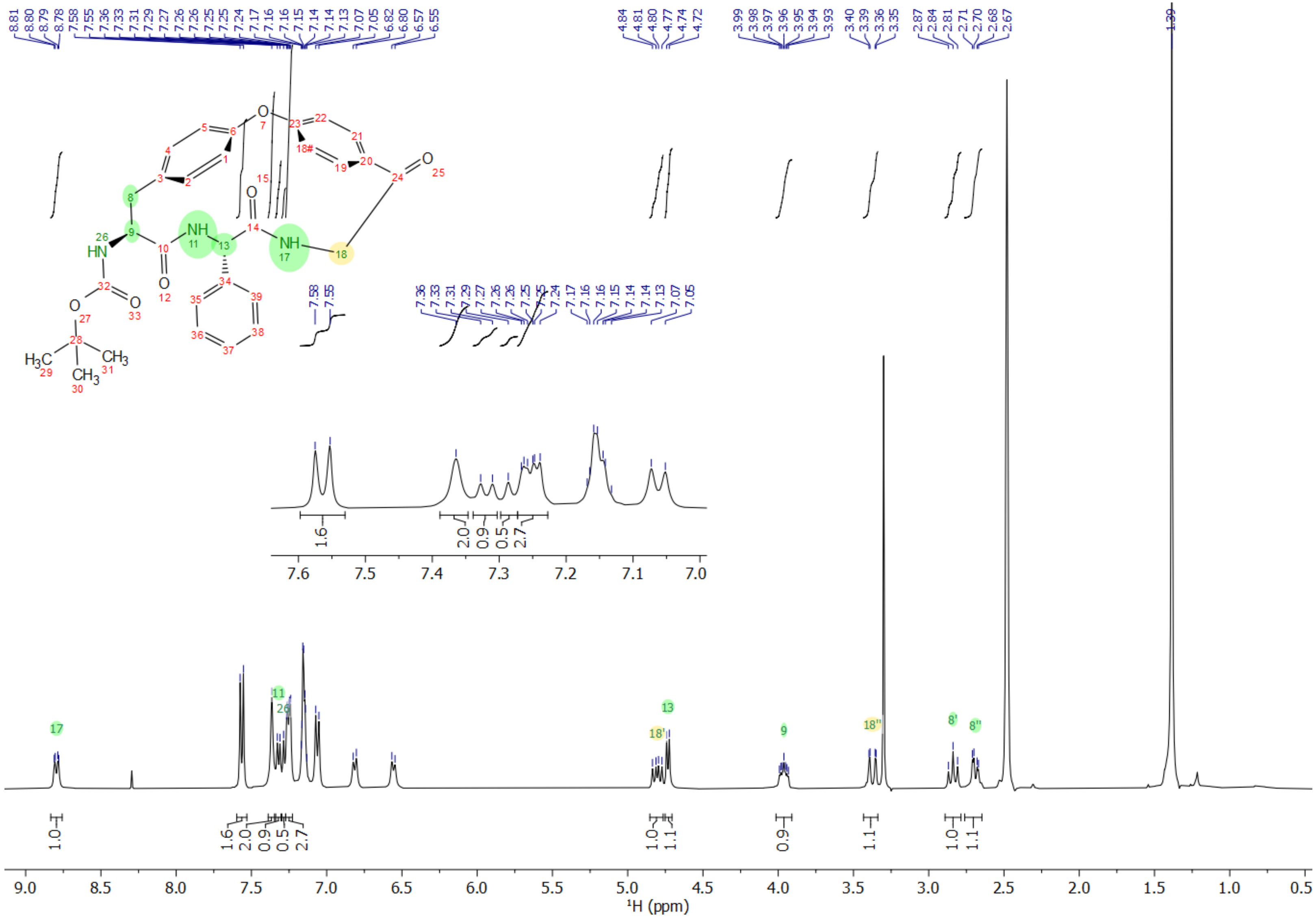

Figure S5. ^{1}H NMR spectrum of compound **2** in DMSO-d_6 recorded at 25 °C at 400 MHz.

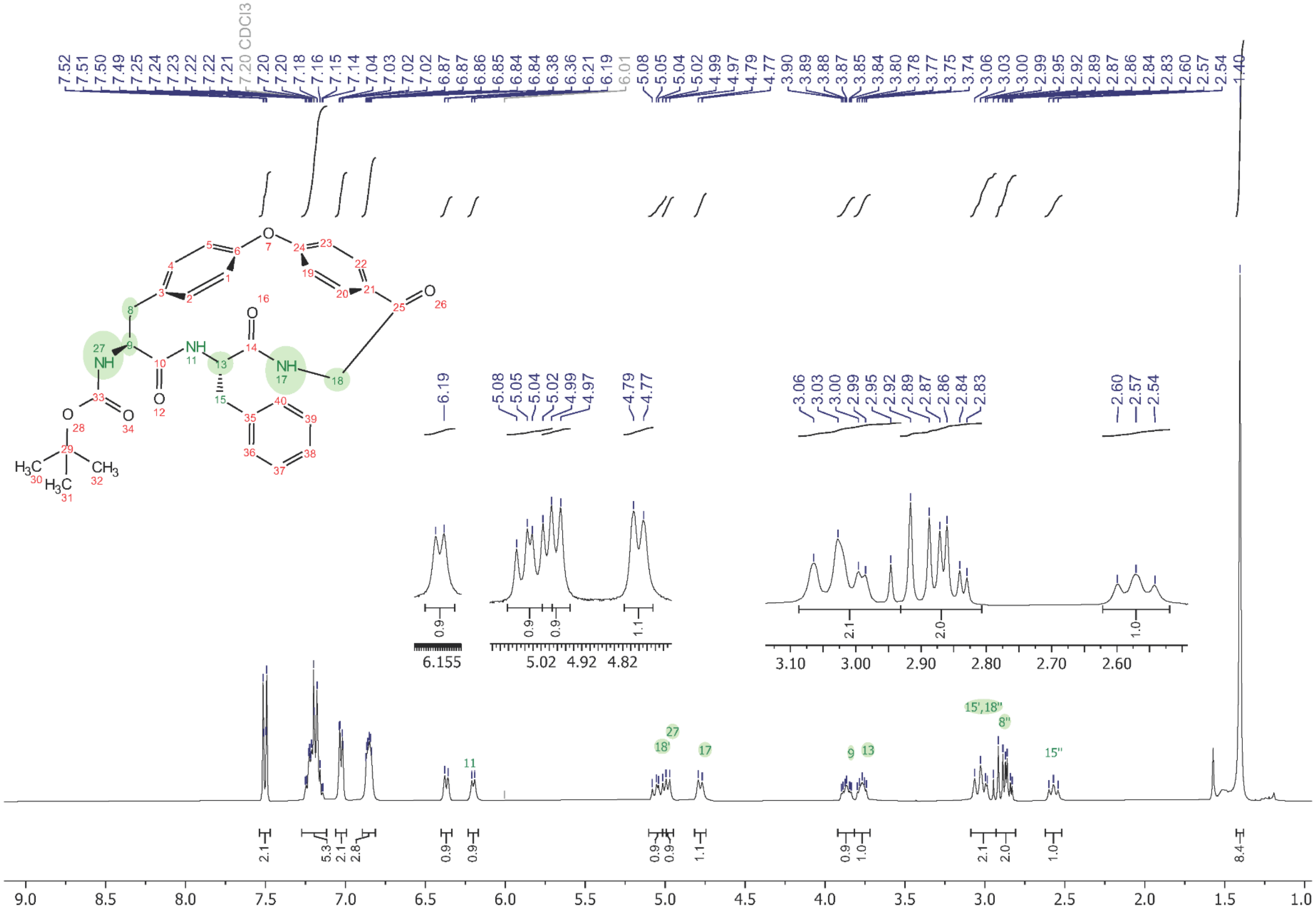

Figure S6. ^1H NMR spectrum of compound **3** in $CDCl_3$ recorded at 25 °C at 400 MHz.

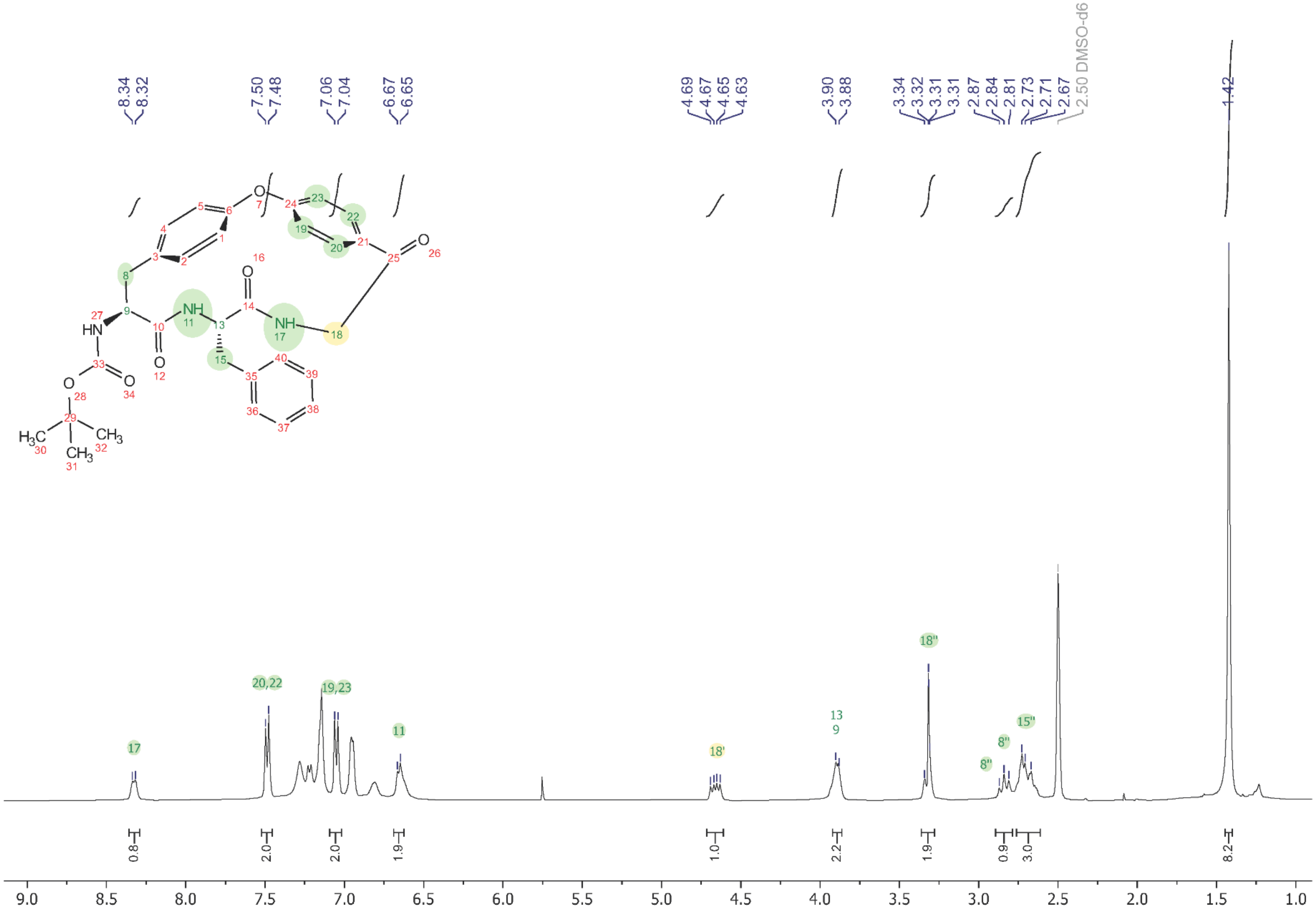

Figure S7. ^{1}H NMR spectrum of compound **3** in DMSO-d_6 recorded at 25 °C at 400 MHz.

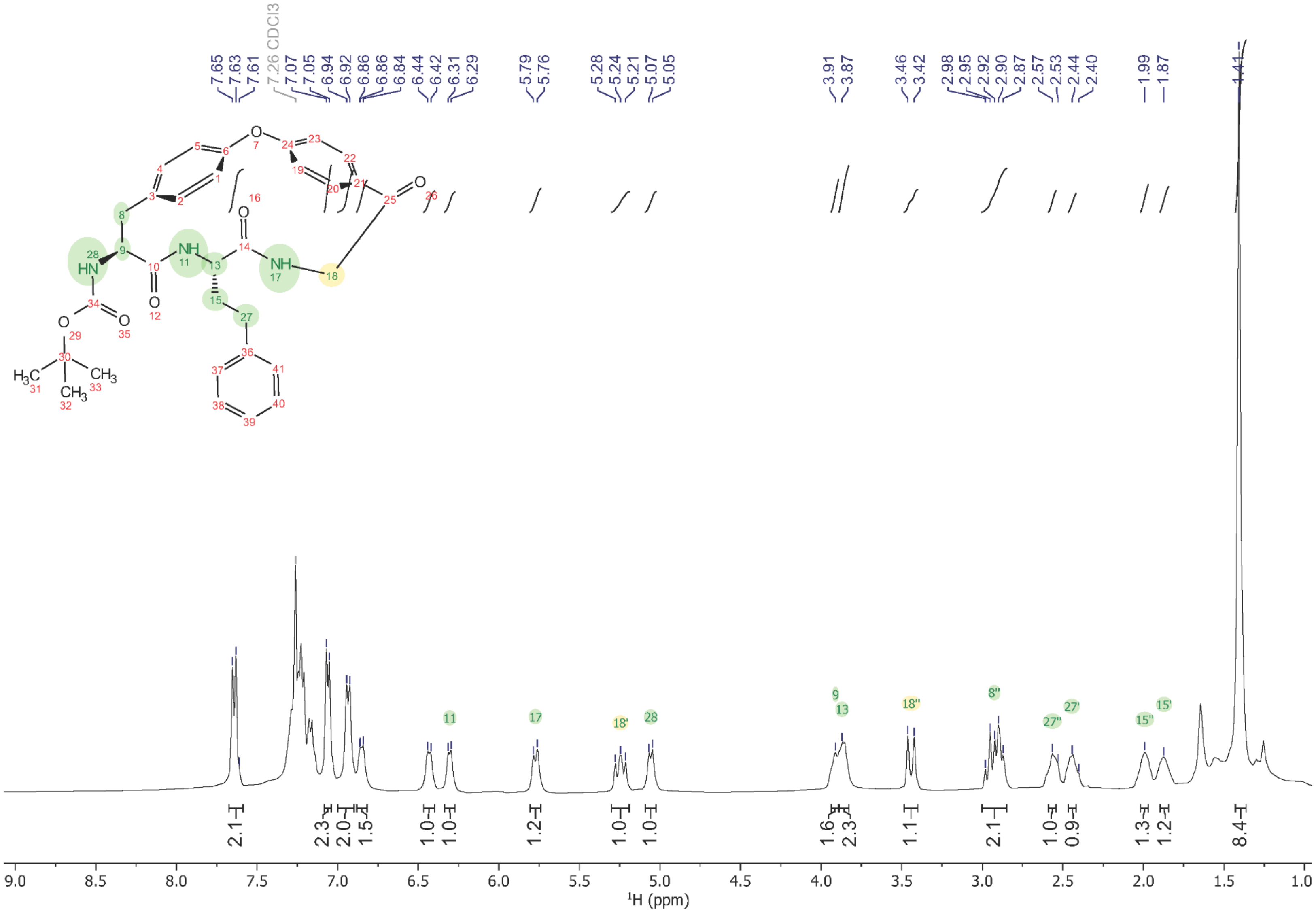

Figure S8. ^{1}H NMR spectrum of compound **4** in $CDCl_3$ recorded at 25 °C at 400 MHz.

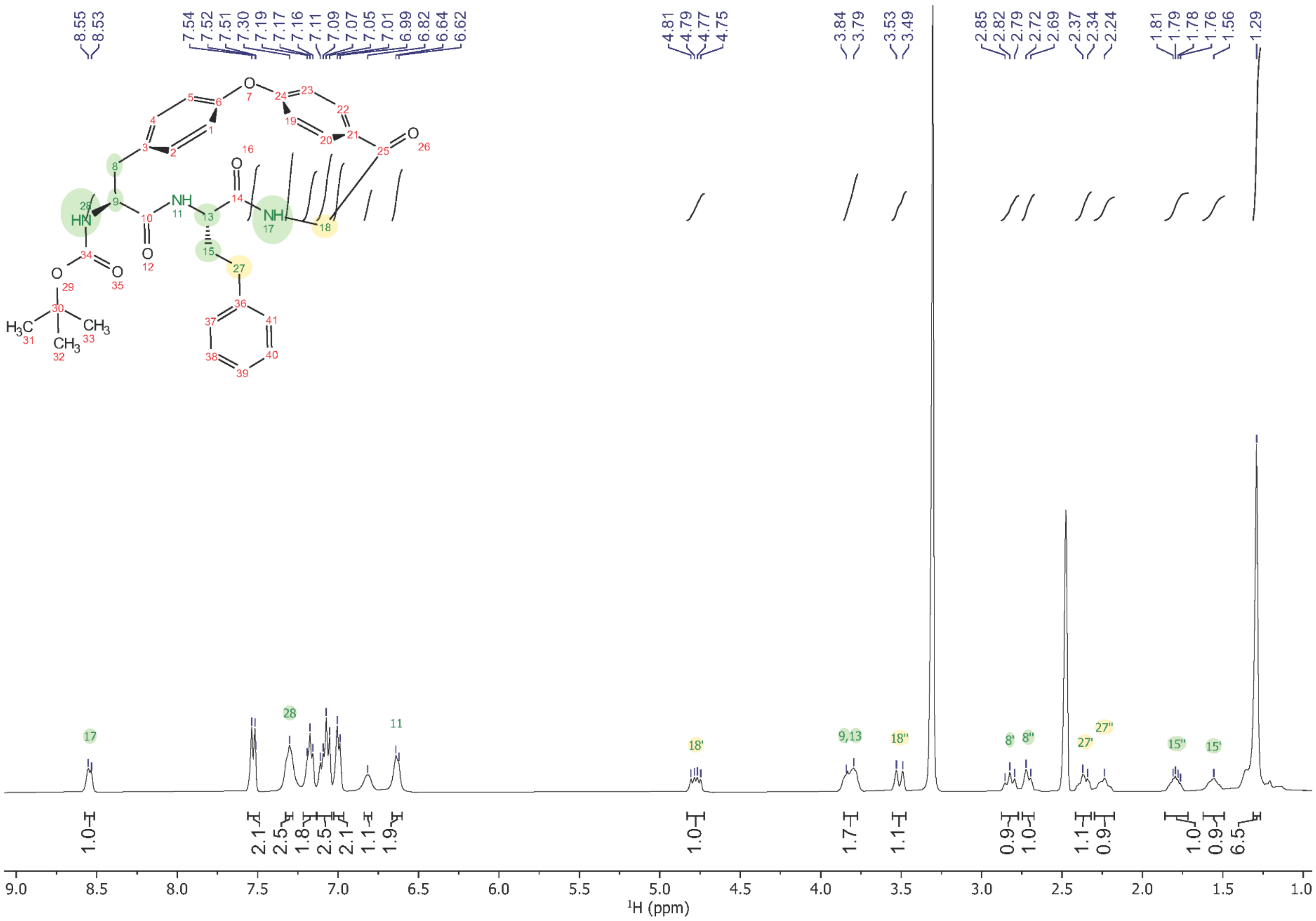

Figure S9. ^{1}H NMR spectrum of compound **4** in DMSO-d_6 recorded at 25 °C at 400 MHz.

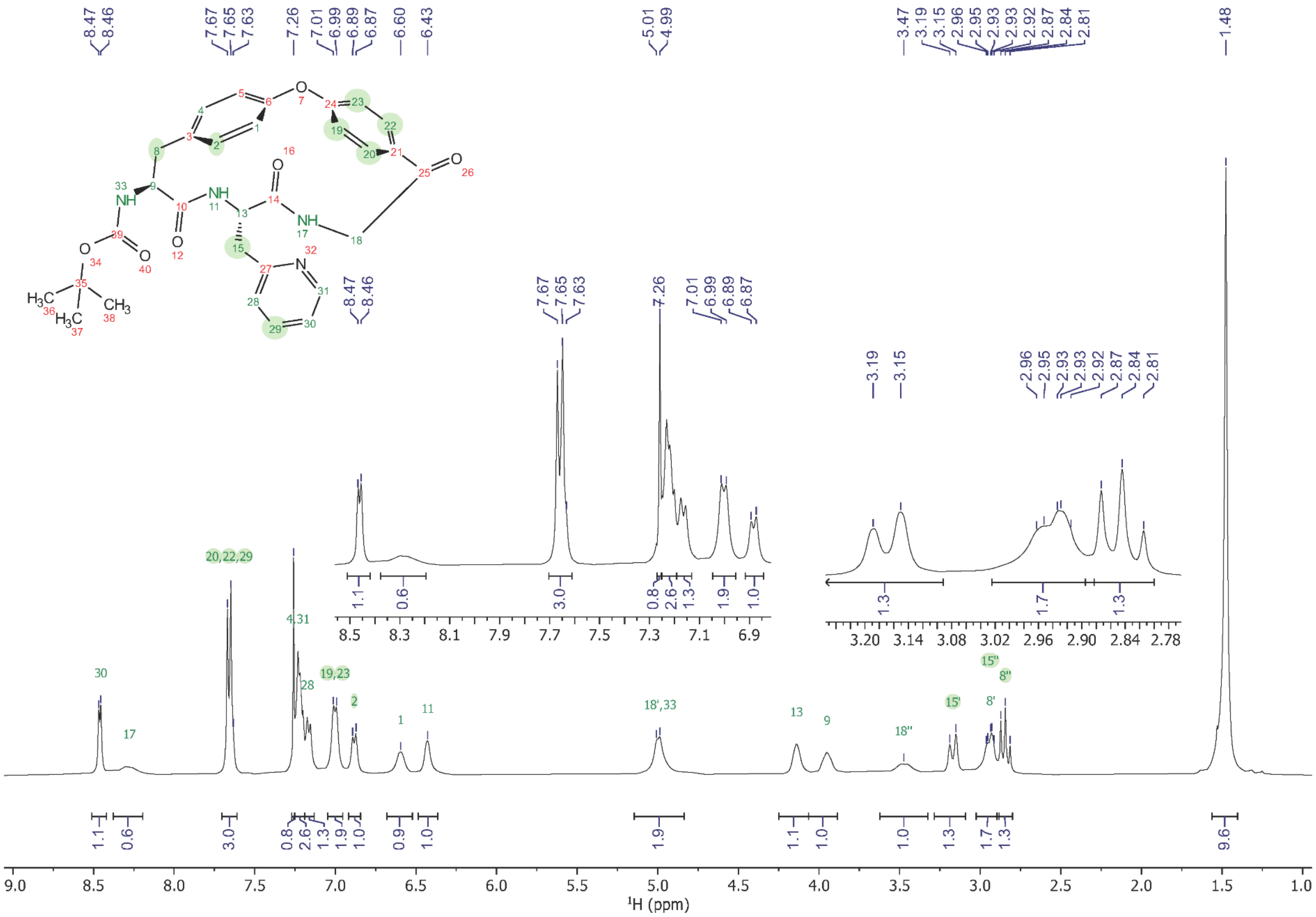

Figure S10. ^{1}H NMR spectrum of compound **5** in $CDCl_3$ recorded at 25 °C at 400 MHz.

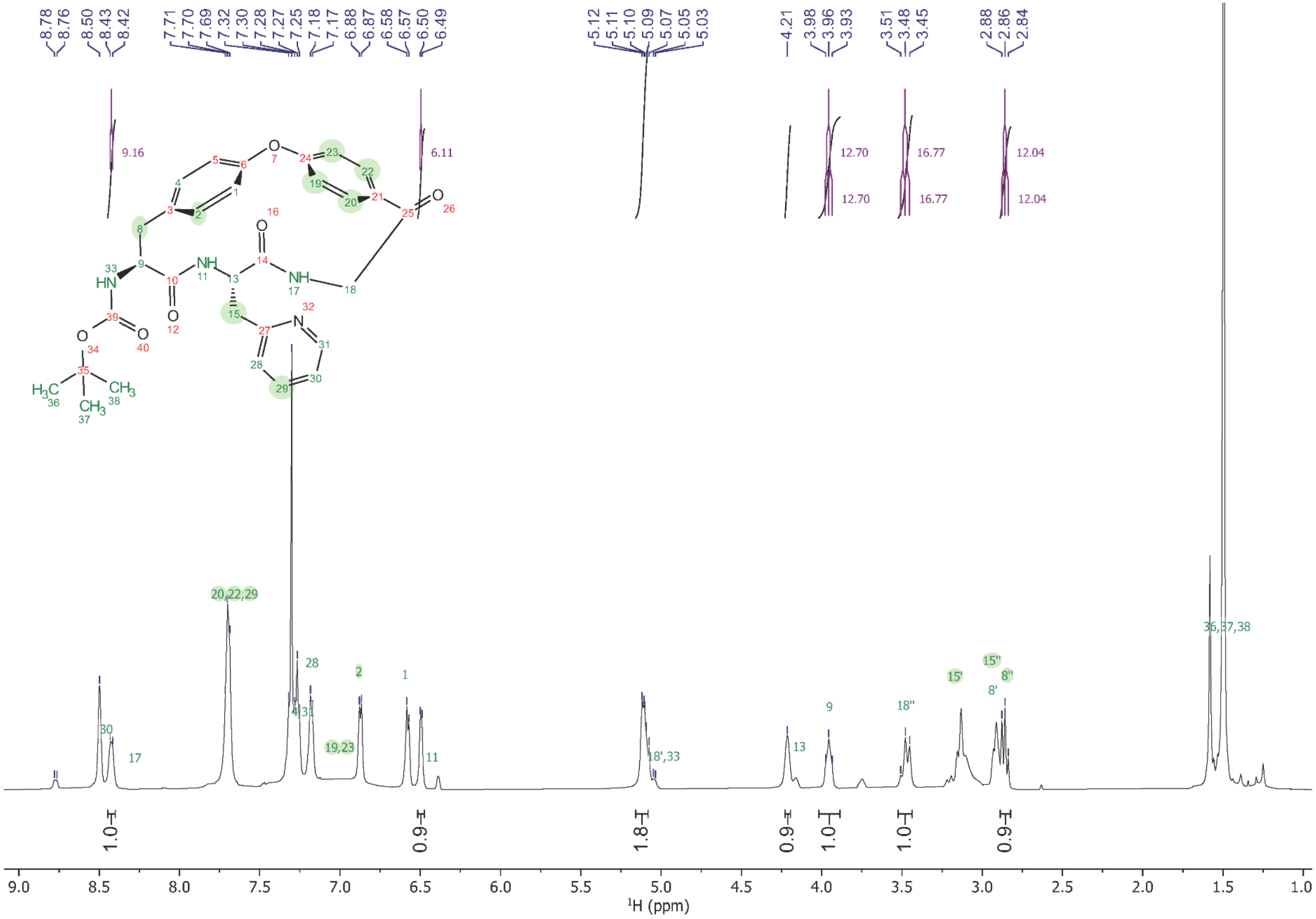

Figure S11. ^{1}H NMR spectrum of compound **5** in $CDCl_3$ at -20 °C at 600 MHz.

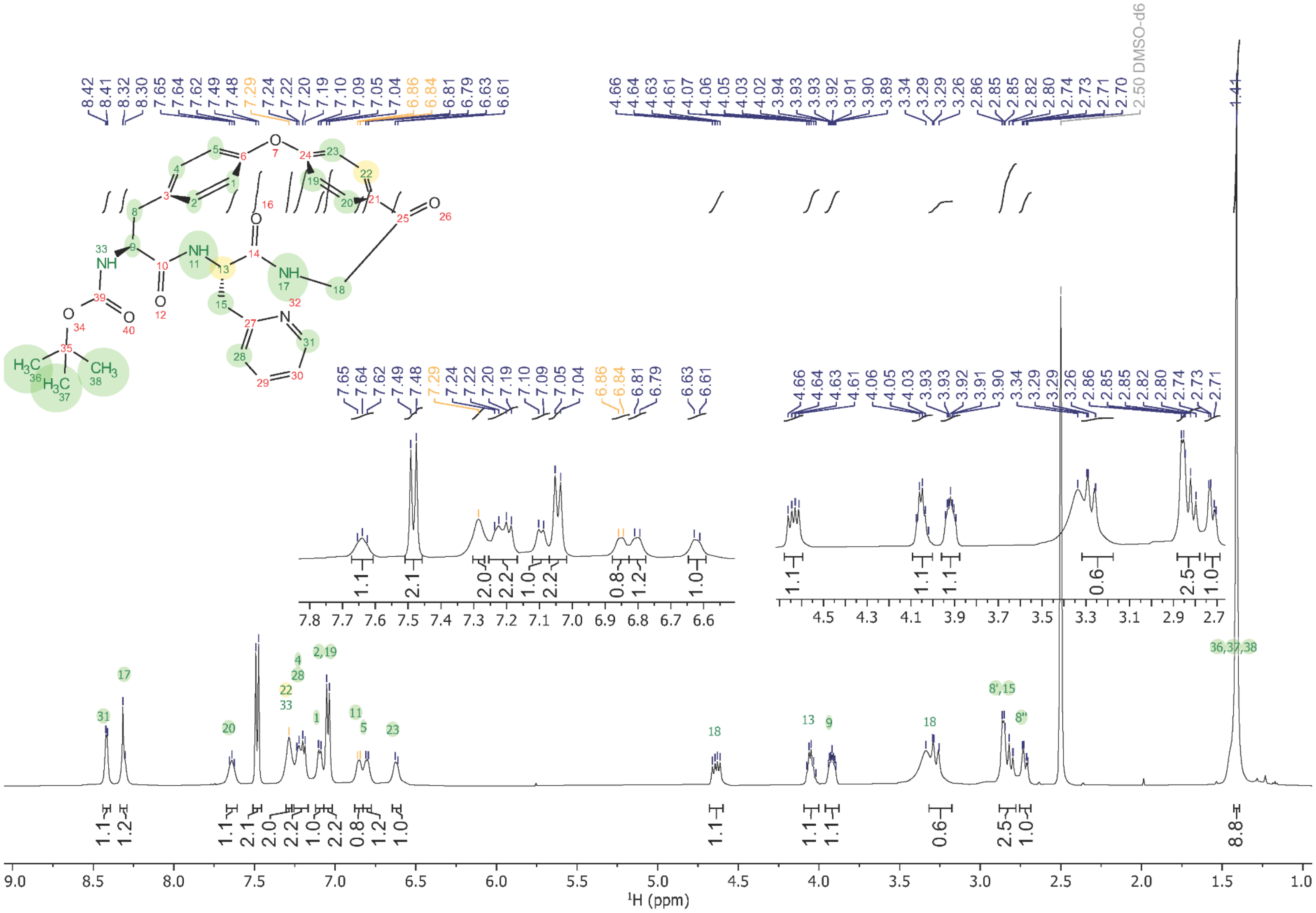

Figure S12. ^{1}H NMR spectrum of compound **5** in DMSO-d_6 recorded at 25 °C at 500 MHz.

Table S1. Summary of chemical shifts (δ, ppm) obtained from NMR spectroscopy and calculations in chloroform.

**Protocol**	**Compound No.**	**Experimental Chemical Shifts (δ, ppm)**			**Predicted Chemical Shifts (Minimum Energy Conformer; δ, ppm)**		
		NH(I)	NH(II)	NH(III)	NH(I)	NH(II)	NH(III)
**MC**	**1**	5.73	6.20	5.01	4.87	6.04	4.83
	**2**	5.63	6.85	4.90	4.95	6.10	4.56
	**3**	4.78	6.20	5.00	3.42	6.28	4.90
	**4**	5.78	6.30	5.06	5.05	5.86	5.00
	**5**	8.29	6.43	4.99	4.39	6.37	4.95
**MC:DFT**	**1**	5.73	6.20	5.01	5.10	5.88	4.74
	**2**	5.63	6.85	4.90	5.07	6.75	4.49
	**3**	4.78	6.20	5.00	3.75	6.06	4.81
	**4**	5.78	6.30	5.06	5.13	6.20	4.94
	**5**	8.29	6.43	4.99	7.09	6.38	4.68
**MD**	**1**	5.73	6.20	5.01	5.41	7.00	5.17
	**2**	5.63	6.85	4.90	4.83(4.32)	5.56 (6.89)	5.32 (4.71)
	**3**	4.78	6.20	5.00	4.38	7.32	4.69
	**4**	5.78	6.30	5.06	6.11	6.76	5.09
	**5**	8.29	6.43	4.99	5.97 (4.65)	4.66 (6.88)	4.38 (5.11)
**MD:DFT**	**1**	5.73	6.20	5.01	5.12	5.88	4.74
	**2**	5.63	6.85	4.90	5.14 (4.71)	6.67 (7.07)	4.52 (4.96)
	**3**	4.78	6.20	5.00	3.74	6.05	4.82
	**4**	5.78	6.30	5.06	4.68	6.04	4.75
	**5**	8.29	6.43	4.99	9.83 (10.89)	6.50 (7.03)	4.83 (4.97)

Note: The calculated shifts of the conformations of compounds **2** and **5** that have the R-substituent in an axial orientation on the macrocyclic ring are shown in parenthesis.

Table S2. Summary of chemical shifts (δ, ppm) obtained from NMR spectroscopy and calculations in DMSO.

Protocol	Compound No.	Experimental Chemical Shifts (*δ*, ppm)			Predicted Chemical Shifts (Minimum Energy Conformer; *δ*, ppm)		
		NH(I)	NH(II)	NH(III)	NH(I)	NH(II)	NH(III)
**MC**	**1**	8.51	6.44	7.33	5.08	5.44	5.06
	**2**	8.79	7.31	7.29	5.33	5.92	4.84
	**3**	8.33	6.66	7.22	3.80	5.63	5.07
	**4**	8.54	6.63	7.30	5.11	5.51	4.90
	**5**	8.31	6.85	7.19	3.68	5.63	5.08
**MC:DFT**	**1**	8.51	6.44	7.33	5.27	6.06	4.82
	**2**	8.79	7.31	7.29	5.34	6.77	4.55
	**3**	8.33	6.66	7.22	3.98	6.08	4.87
	**4**	8.54	6.63	7.30	5.51	5.89	4.69
	**5**	8.31	6.85	7.19	9.81	6.54	4.88
**MD**	**1**	8.51	6.44	7.33	9.82	6.77	4.71
	**2**	8.79	7.31	7.29	6.73 (3.97)	6.73 (5.40)	7.42 (5.29)
	**3**	8.33	6.66	7.22	6.65	5.90	6.67
	**4**	8.54	6.63	7.30	5.62	8.45	7.68
	**5**	8.31	6.85	7.19	4.83 (4.40)	5.22 (6.75)	4.43 (6.33)
**MD:DFT**	**1**	8.51	6.44	7.33	9.29	6.68	8.93
	**2**	8.79	7.31	7.29	10.29 (4.75)	6.86 (6.83)	9.14 (8.33)
	**3**	8.33	6.66	7.22	9.25	6.37	9.05
	**4**	8.54	6.63	7.30	9.34	8.64	8.94
	**5**	8.31	6.85	7.19	9.27 (10.66)	6.49 (8.09)	9.15 (8.44)

Note: The calculated shifts of the conformations of compounds **2** and **5** that have the R-substituent in an axial orientation on the macrocyclic ring are shown in parenthesis.

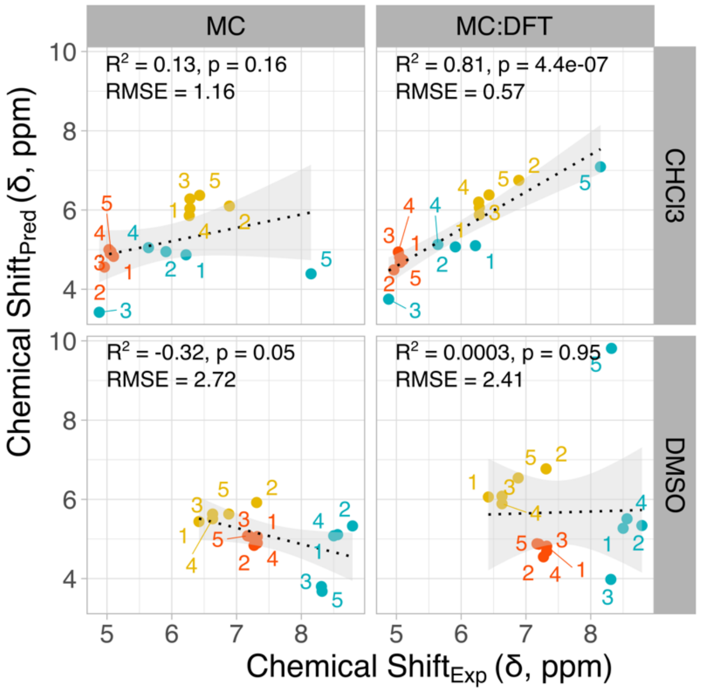

Figure S13. Correlation between experimental chemical shifts and predicted chemical shifts of the minimum energy conformer (MEC) using an implicit solvation model (**MC** refers to the MCMM and **MC:DFT** corresponds to the MCMM results followed by DFT optimizations). The chemical shifts of each of the three amide protons NH-I (red), NH-II (green), and NH-III (blue) are indicated.

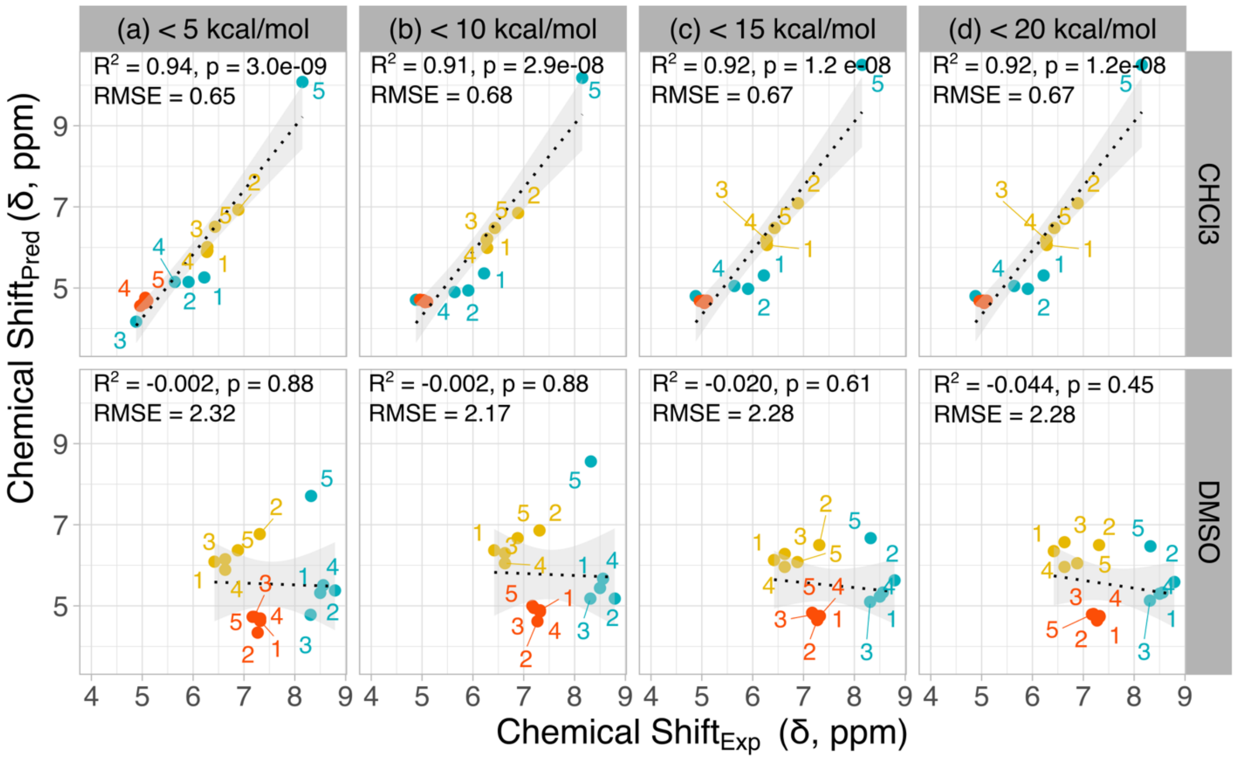

Figure S14. Summary of correlations between experimental and the mean chemical shifts predicted using an implicit solvation model with protocol – **MC:DFT$_{ens}$**. The mean chemical shifts were obtained by averaging all chemical shifts of different conformational ensembles below 5, 10,

15, and 20 kcal mol-1. The chemical shifts of each of the three amide protons NH-I (red), NH-II (green), and NH-III (blue) are indicated.

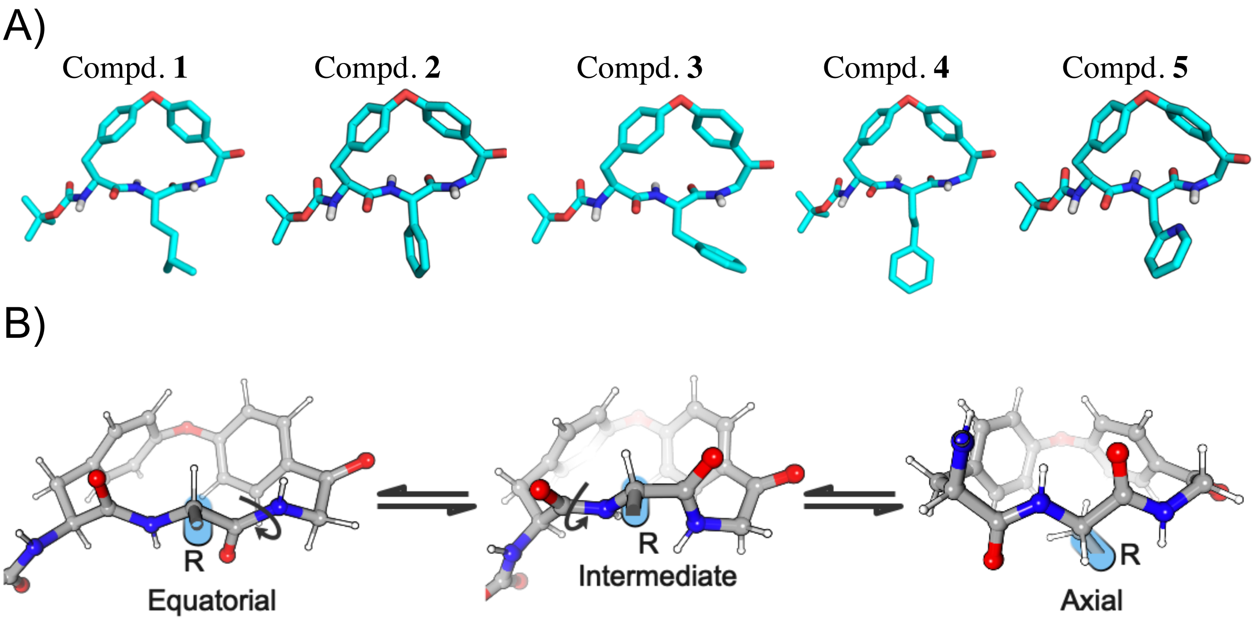

Figure S15. A) Minimum energy conformers (MECs) obtained by protocol – **MC:DFT** for macrocycles **1-5** in implicit chloroform showing that R side-chain oriented equatorially. **B)** Schematic description of the conformational interconversion of the macrocyclic ring that results in reorientation of the R side-chain from and equatorial to an axial position.

Table S3. The Gibbs free energies of the conformers obtained by protocol **MC:DFT** for macrocycles **1-5** in implicit chloroform. The Gibbs free energies are given relative to the minimum energy conformers in kcal mol^{-1}.*

Conformer	Compd. 1	Compd. 2	Compd. 3	Compd. 4	Compd. 5
**1**	0.00 (eq.)	0.00 (eq.)	0.00 (eq.)	0.00 (eq.)	0.00 (eq.)
**2**	0.79 (eq.)	1.56 (eq.)	0.82 (eq.)	1.15 (eq.)	2.98 (ax.)
**3**	1.70 (eq.)	6.31 (ax.)	1.75 (eq.)	3.50 (eq.)	3.14 (ax.)
**4**	2.10 (eq.)	6.32 (ax.)	2.67 (eq.)	3.94 (eq.)	4.59 (ax.)
**5**	2.54 (eq.)	8.36 (ax.)	4.55 (eq.)	4.26 (eq.)	4.59 (ax.)
**6**	4.67 (eq.)	8.52 (ax.)	6.60 (ax.)	4.56 (eq.)	4.59 (ax.)
**7**	5.22 (eq.)	8.53 (ax.)	8.40 (ax.)	9.75 (ax.)	5.36 (ax.)
**8**	5.80 (eq.)	10.58 (ax.)	8.49 (ax.)	9.82 (ax.)	5.41 (ax.)
**9**	9.17 (ax.)	13.81 (ax.)	8.94 (ax.)	12.68 (ax.)	7.23 (ax.)
**10**	11.39 (ax.)	21.59 (ax.)	10.84 (ax.)	22.06 (ax.)	7.96 (ax.)

*eq.: equatorial and ax.: axial conformers

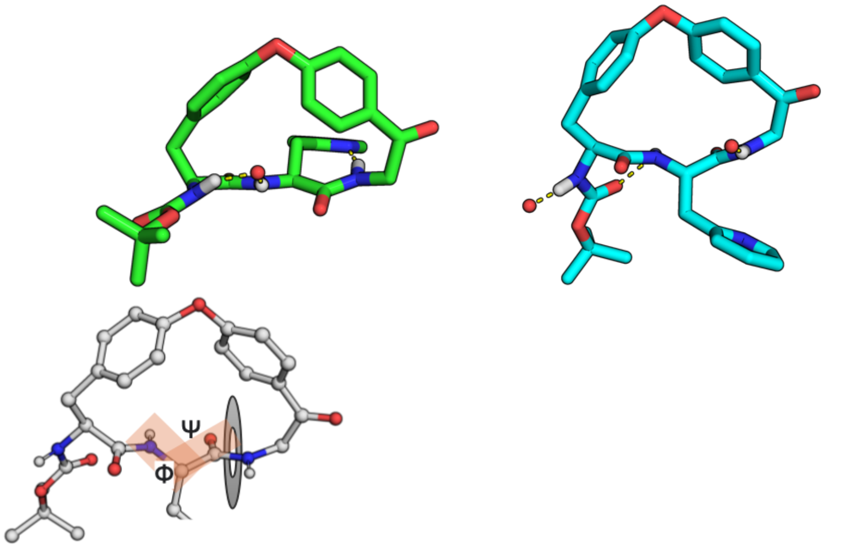

Axial conformation **Equatorial conformation**

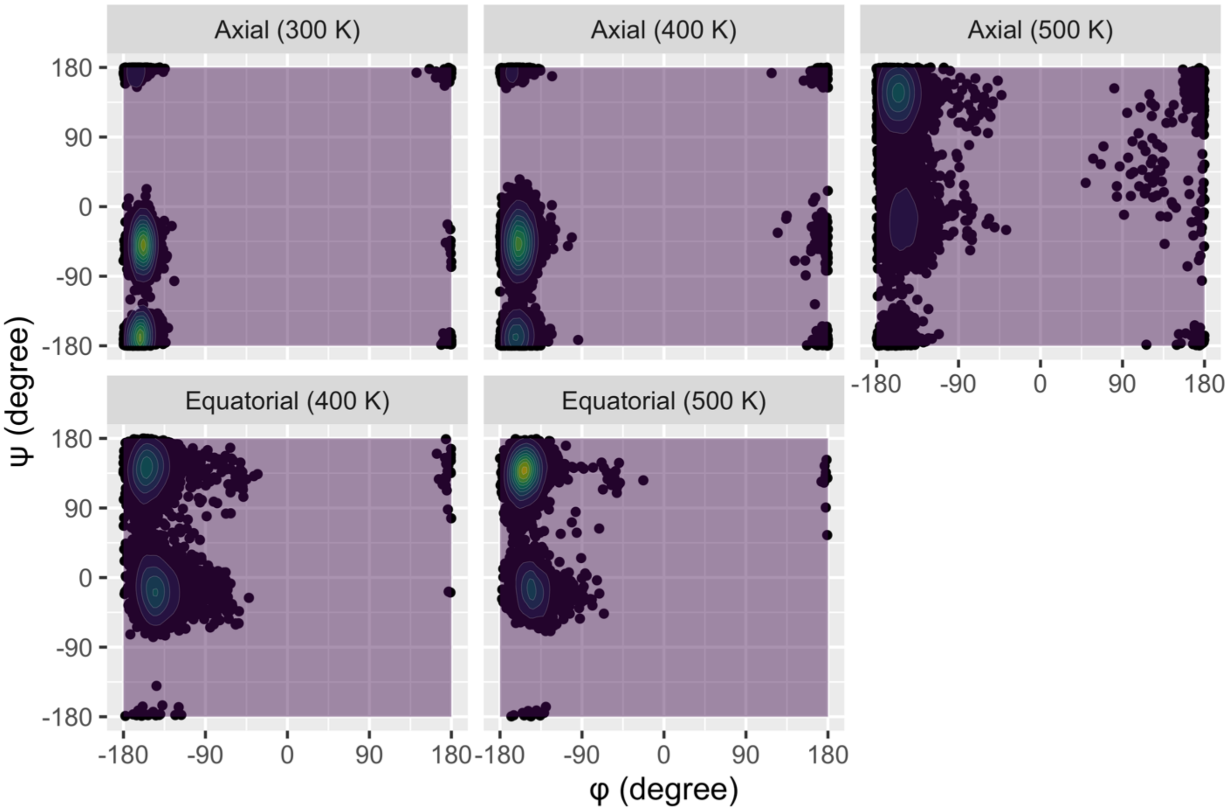

Figure S16. Density plots of torsion angles (ϕ, ψ) throughout a 50 ns MD simulation for compound **5** in explicit DMSO at 300, 400, and 500K. Simulations were started from

conformations that had R side chain in an axial (top panels) or an equatorial orientation (bottom panels). DMSO molecules are represented as red spheres in the structures above the density plots. The ϕ, ψ angles are defined in the structure at the top right.

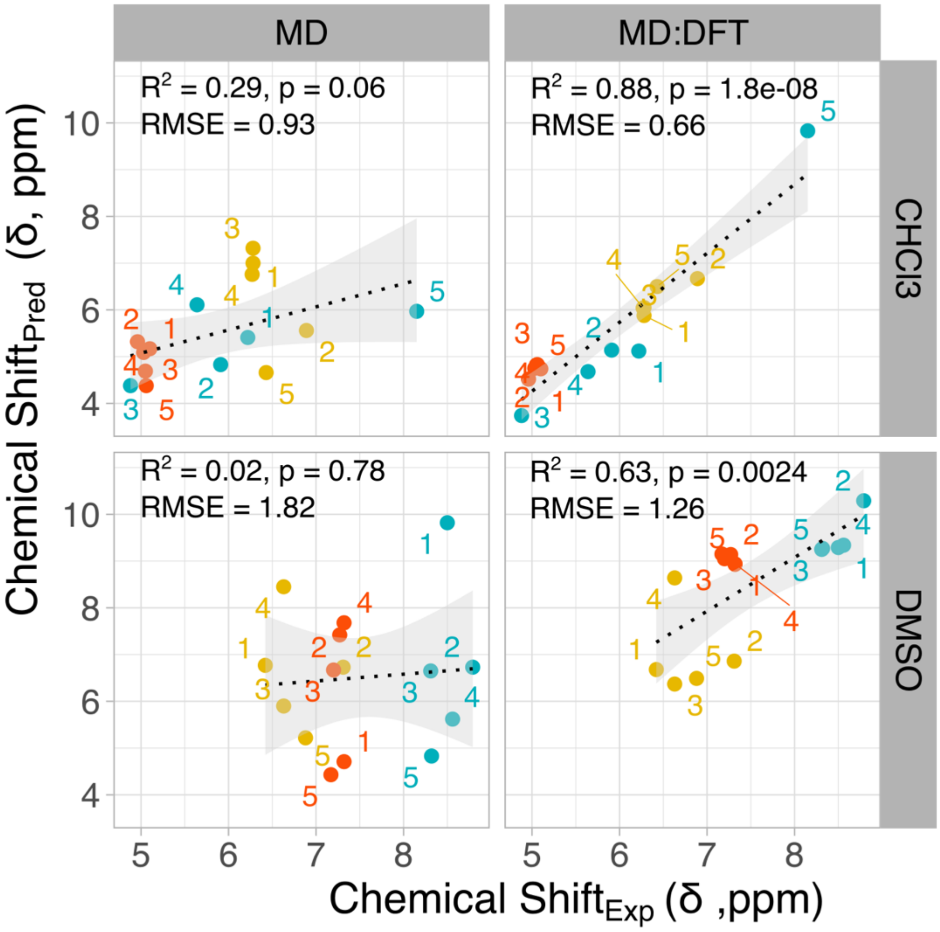

Figure S17. Correlations between experimental chemical shifts and predicted chemical shifts of minimum energy conformations (MEC) using explicit solvation models. **MD** refers to the molecular dynamics simulations and **MD:DFT** corresponds to the MD results followed by DFT optimizations. All MEC conformations have the R side chain in an equatorial orientation. The chemical shifts of each of the three amide protons NH-I (red), NH-II (green), and NH-III (blue) are indicated.

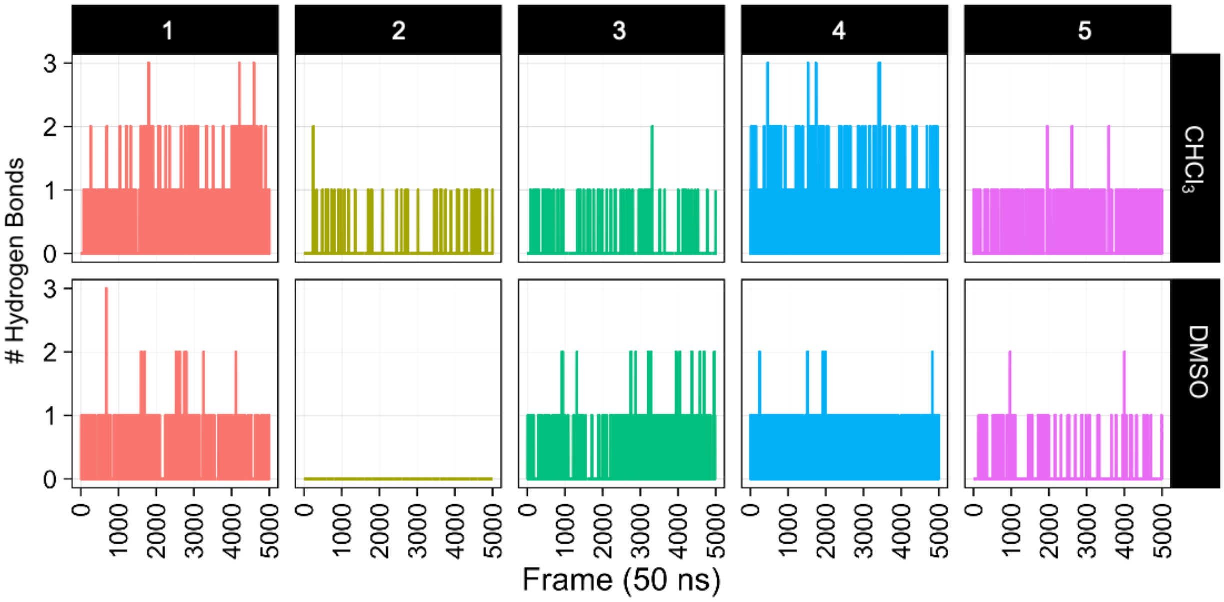

Figure S18. Number of ***intra***molecular hydrogen bonds (IMHB) of compounds **1-5** throughout 50 ns MD simulations in explicit chloroform and DMSO.

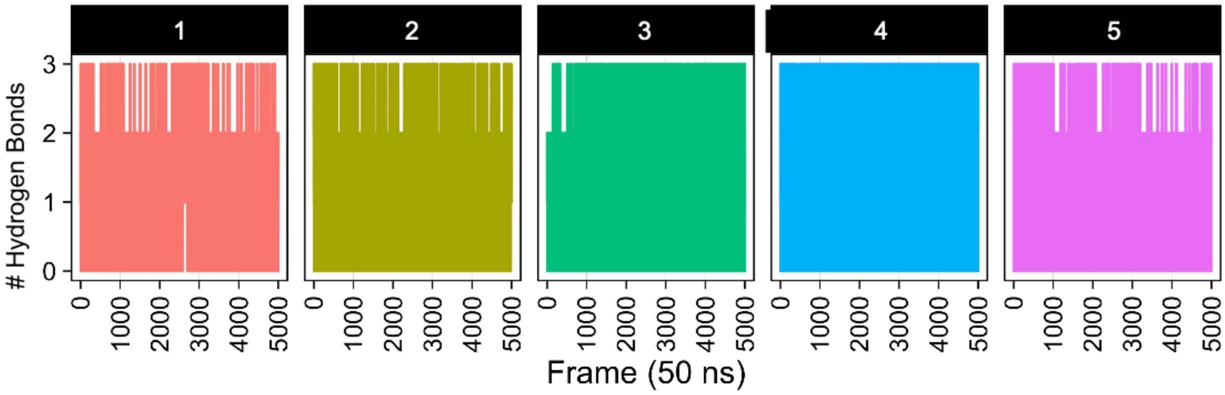

Figure S19. Number of ***inter***molecular hydrogen bonds between DMSO and compounds **1-5** throughout 50 ns MD simulations in DMSO.

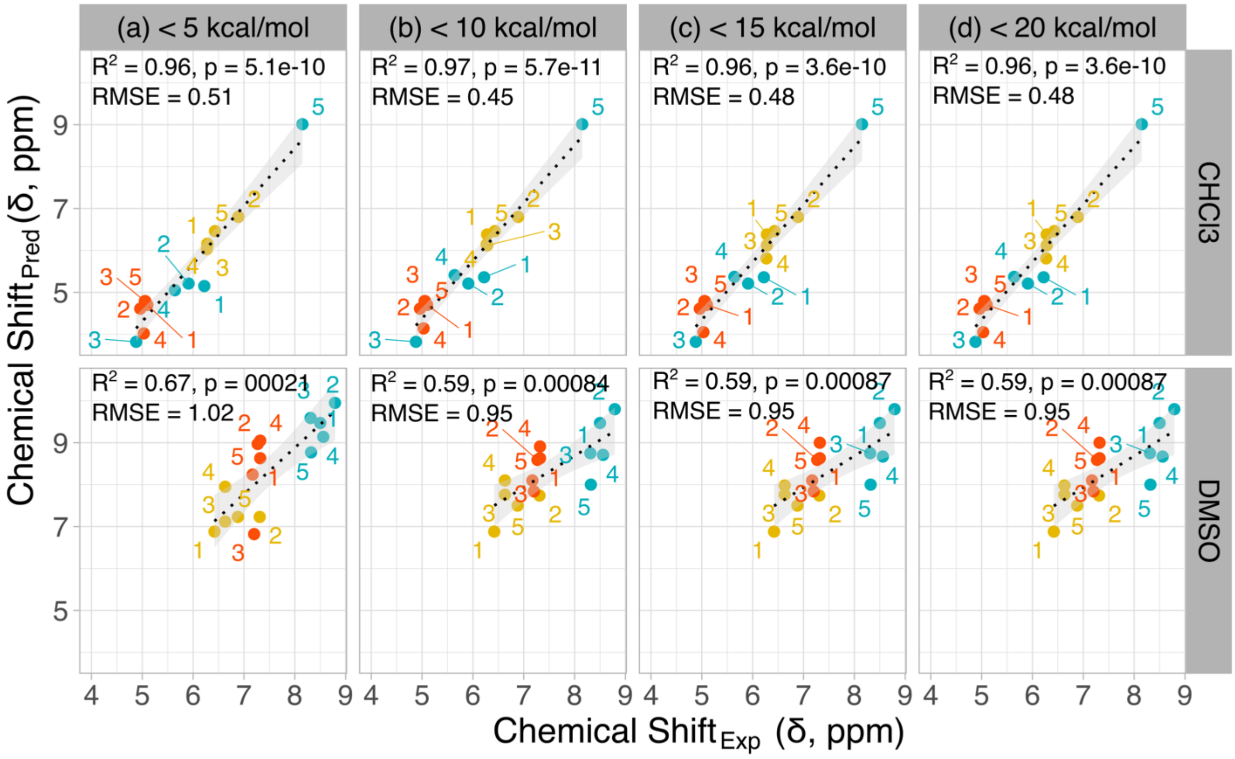

Figure S20. Summary of the correlations between experimental and mean chemical shifts predicted using an explicit solvation model with protocol **MD:DFT$_{ens}$**. The mean chemical shifts were obtained by averaging all chemical shifts of different conformational ensembles below 5, 10, 15, and 20 kcal mol^{-1}. The chemical shifts of each of the three amide protons NH-I (red), NH-II (green), and NH-III (blue) are indicated.

Table S4. Comparison of $^{3}J_{H,H}$ coupling constants calculated for the MECs of protocols **MC:DFT**, **MD** and **MD:DFT** to those determined by NMR spectroscopy in $CDCl_3$ for compound **1-5**[a]

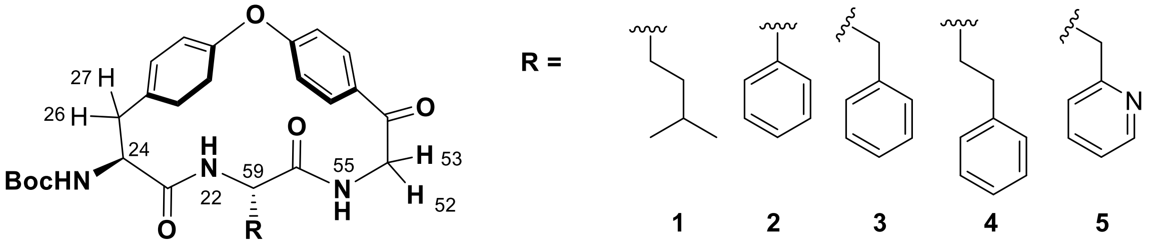

Cpd.	Protocol	J (Hz) NH-I 55-52	J (Hz) NH-I 55-53	J (Hz) NH-II 22-59	J (Hz) H-24 24-26	J (Hz) H-24 24-27
1	MC:DFT	10.3 (173.6°)	2.4 (54.9°)	6.0 (144.6°)	9.9 (178.5°)	4.1 (61.8°)
	MD	10.3 (-176.1°)	1.1 (59.3°)	8.8 (166.3°)	10.0 (-164.8°)	2.1 (78.5°)
	MD:DFT	10.3 (173.5°)	2.4 (54.8°)	6.1 (145.1°)	9.9 (178.6°)	4.1 (61.9°)
	**Exp.**	10.4	2.3	7.7	10.4	4.1
2	MC:DFT	10.2 (172.2°)	2.5 (53.3°)	4.3 (131.2°)	9.8 (177.4°)	4.4 (60.8°)
	MD	8.5 (-160.9°)	0.2 (85.8°)	1.5 (110.7°)	10.5 (178.5°)	3.9 (73.5°)
	MD:DFT	10.2 (172.6°)	2.5 (53.7°)	4.3 (131.4°)	9.9 (177.5°)	4.4 (60.9°)
	**Exp.**[b]	10.5	br	6.6	10.3	4.8
3	MC:DFT	10.1 (168.5°)	3.0 (49.7°)	5.5 (140.3°)	9.9 (178.1°)	4.2 (61.4°)
	MD	10.3 (174.8°)	1.5 (64.3°)	8.1 (142.5°)	14.1 (-169.4°)	2.8 (73.6°)
	MD:DFT	10.1 (168.5°)	3.0 (49.7°)	5.5 (140.3°)	9.9 (178.1°)	4.2 (61.4°)
	**Exp.**	10.2	br	7.4	10.5	4.3
4	MC:DFT	2.2 (55.8°)	10.3 (174.5°)	4.5 (132.3°)	9.9 (176.7°)	4.4 (60.1°)
	MD	0.1 (-96.2°)	8.9 (143.8°)	7.4 (-171.7°)	8.2 (170.8°)	1.6 (76.6°)
	MD:DFT	2.2 (55.4°)	10.4 (174.2°)	7.6 (155.3°)	11.1 (-179.5°)	3.7 (62.9°)
	**Exp.**	2.4	10.3	7.6	12.0	4.3
5	MC:DFT	2.9 (48.8°)	10.3 (167.8°)	6.7 (147.6°)	11.0 (179.5°)	3.8 (62.1°)
	MD	8.8 (168.3°)	3.8 (51.4°)	3.3 (121.6°)	11.2 (-172.9°)	3.5 (67.7°)
	MD:DFT	9.8 (-171.4°)	1.0 (69.5°)	9.2 (165.0°)	11.3 (179.7°)	3.9 (62.1°)
	**Exp.**[b]	9.2	br	6.1	10.5	ol

[a]Cut-offs for the agreement between calculated and experimentally determined coupling constants: High: ≤ ±1 Hz; Moderate: ± (1–2) Hz; Low: ≥ ±2 Hz. Moderate and Low agreement has been marked in orange and red, respectively.
[b]Determined at -20 °C
Abbreviations: br: broad, ol: overlap

Table S5. Comparison of $^{3}J_{H,H}$ coupling constants calculated for the MECs of protocols **MC:DFT**, **MD** and **MD:DFT** to those determined by NMR spectroscopy in DMSO-d_6 for compound **1-5**[a]

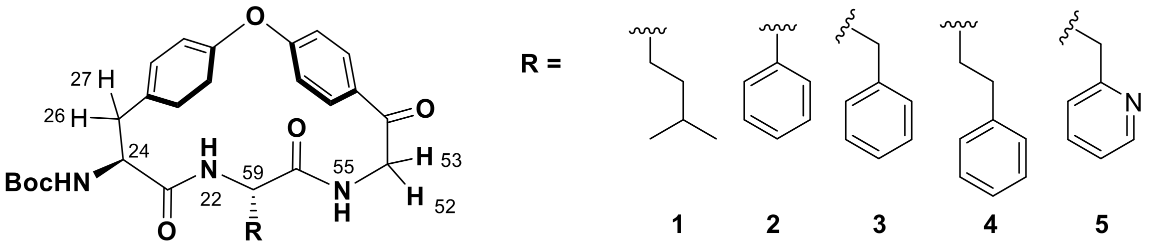

Cpd.	Protocol	J (Hz) NH-I 55-52	J (Hz) NH-I 55-53	J (Hz) NH-II 22-59	J (Hz) H-24 24-26	J (Hz) H-24 24-27
1	MC:DFT	10.0 (172.3°)	2.5 (53.4°)	4.6 (132.9°)	9.9 (176.8°)	4.5 (60.2°)
	MD	10.7 (179.8°)	1.6 (61.4°)	8.1 (-172.4°)	13.0 (-173.3°)	3.3 (71.2°)
	MD:DFT	9.8 (175.1°)	2.2 (56.2°)	10.3 (175.8°)	11.2 (-178.7°)	3.6 (129.1°)
	Exp.	8.5	br	7.8	12.0	2.7
2	MC:DFT	10.0 (172.6°)	2.5 (53.7°)	4.3 (131.4°)	9.9 (177.5°)	4.4 (60.9°)
	MD	7.9 (-166.2°)	1.9 (54.1°)	8.1 (159.1°)	10.6 (174.4°)	5.0 (54.75°)
	MD:DFT	9.8 (179.9°)	1.9 (60.2°)	6.0 (140.3°)	11.2 (179.1°)	3.9 (62.2°)
	Exp.	9.4	2.6	7.0	11.9	3.8
3	MC:DFT	10.1 (168.5°)	2.9 (49.7°)	5.7 (140.3°)	10.0 (178.1°)	4.2 (61.4°)
	MD	9.8 (174.8°)	1.0 (64.3°)	6.0 (142.5°)	11.9 (-169.4°)	3.9 (73.6°)
	MD:DFT	9.8 (168.5°)	2.2 (49.7°)	10.2 (140.3°)	11.3 (178.1°)	3.6 (61.4°)
	Exp.	8.2	3.3	7.6	12.1	ol

4	MC:DFT	10.1 (173.9°)	2.4 (55.3°)	6.1 (145.3°)	10.0 (178.8°)	4.1 (62.2°)
	MD	6.8 (-100.0°)	6.5 (142.7°)	10.2 (156.7°)	9.6 (-176.5°)	1.9 (66.7°)
	MD:DFT	2.1 (-57.5°)	9.5 (-175.8°)	10.3 (178.9°)	11.2 (-178.3°)	3.7 (64.4°)
	**Exp.**	3.3	8.5	7.6	12.0	4.2
5	MC:DFT	9.6 (166.3°)	3.1 (46.9°)	3.6 (124.1°)	9.9 (176.0°)	4.6 (59.5°)
	MD	1.1 (-62.0°)	9.9 (-179.6°)	8.3 (164.2°)	10.9 (-163.9°)	1.7 (79.8°)
	MD:DFT	9.9 (-171.4°)	1.0 (69.5°)	9.2 (165.0°)	11.3 (179.7°)	3.9 (62.1°)
	**Exp.**	9.2	br	6.1	10.5	ol

[a]Cut-offs for the agreement between calculated and experimentally determined coupling constants: High: $\leq \pm 1$ Hz; Moderate: $\pm$ (1–2) Hz; Low: $\geq \pm 2$ Hz. Moderate and Low agreement has been marked in orange and red, respectively.

Abbreviations: br: broad, ol: overlap

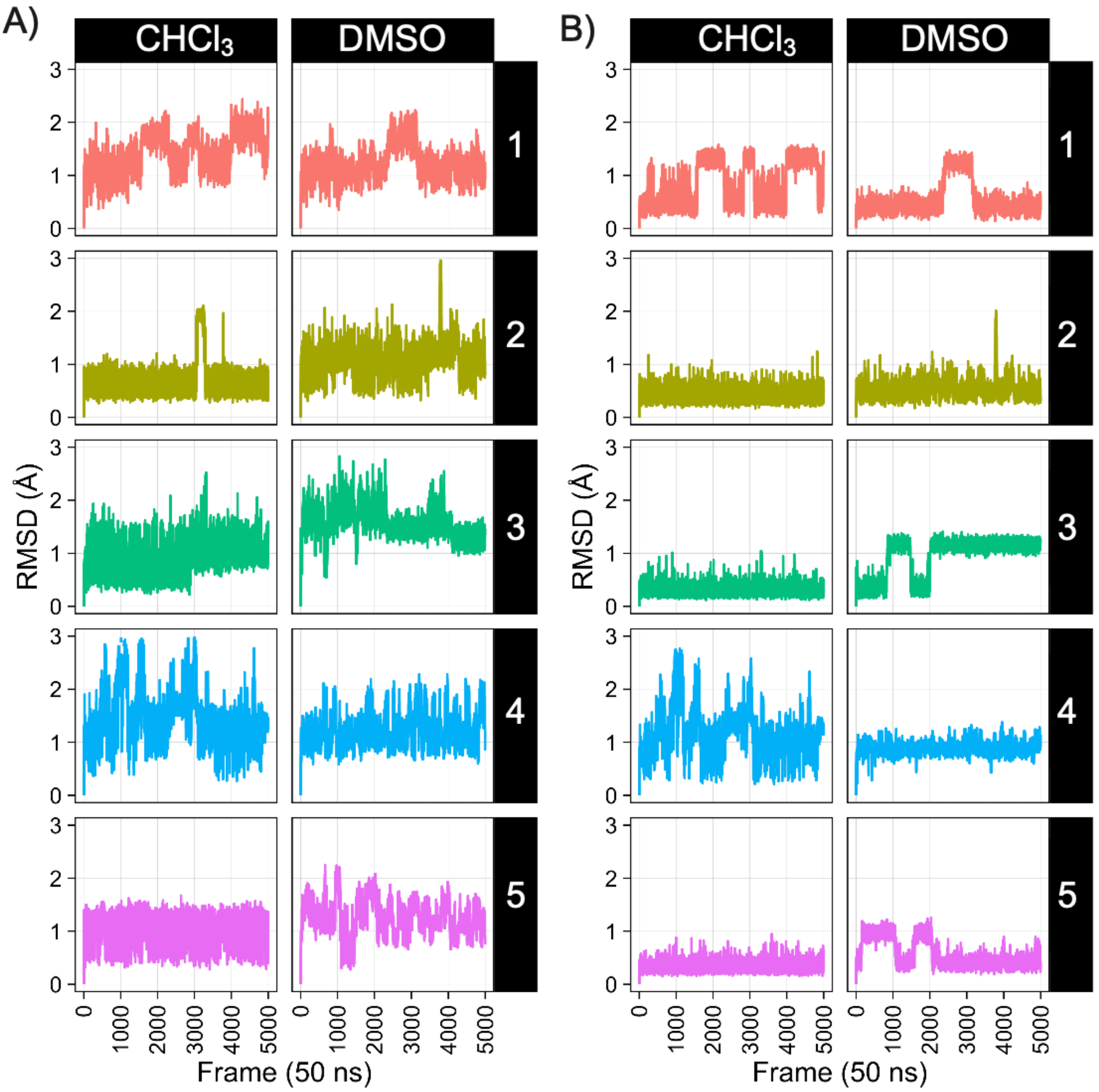

Figure S21. Root-mean-square-deviation (RMSD) plot of the macrocyclic ring and side chains **(A)**, and only the macrocyclic ring **(B)** in apolar and polar environments. The RMSD was calculated as the deviation from the initial geometry by considering all heavy atoms.

Table S6. The Gibbs free energies of state 1 and state 2 conformations of macrocycles **1**, **3**, and **5** in chloroform obtained by protocol **MD:DFT** in kcal mol^{-1}.

Cpd.	State 1	State 2
1	0.00	23.74
3	0.00	11.45
5	0.00	5.87

Table S7. Comparison of $^{3}J_{H,H}$ coupling constants calculated for the state 1 and state 2 conformations of the MEC from protocol **MD:DFT** to those determined by NMR spectroscopy in $CDCl_3$ for compound **1**, **3**, and **5**[a,b]

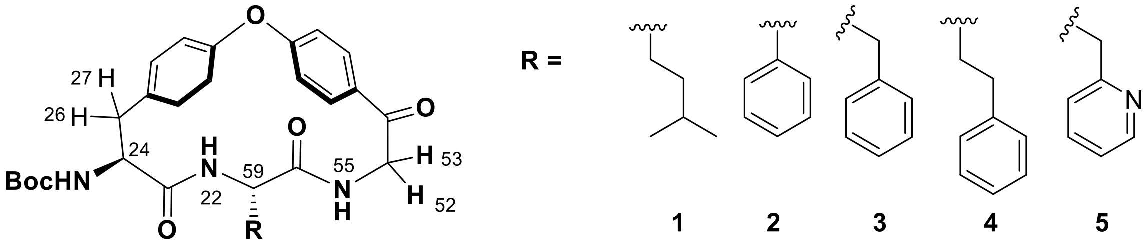

Cpd.	Protocol	J (Hz) NH-I 55-52	J (Hz) NH-I 55-53	J (Hz) NH-II 22-59	J (Hz) H-24 24-26	J (Hz) H-24 24-27
1	State 1	10.3 (173.5°)	2.4 (54.8°)	6.1 (145.1°)	9.9 (178.6°)	4.1 (61.9°)
	State 2	6.2 (-97.4°)	7.2 (145.1°)	10.3 (-173.8°)	11.2 (-178.1°)	3.5 (63.9°)
	**Exp.**	10.4	2.3	7.7	10.4	4.1
3	State 1	10.1 (168.5°)	3.0 (49.7°)	5.5 (140.3°)	9.9 (178.1°)	4.2 (61.4°)
	**Exp.**	10.2	br	7.4	10.5	4.3
5	State 1	9.8 (-171.4°)	1.0 (69.5°)	9.2 (165.0°)	11.3 (179.7°)	3.9 (62.1°)
	**Exp.**[c]	9.2	br	6.1	10.5	ol

[a]Cut-offs for the agreement between calculated and experimentally determined coupling constants: High: $\leq \pm 1$ Hz; Moderate: $\pm$ (1–2) Hz; Low: $\geq \pm 2$ Hz. Moderate and Low agreement has been marked in orange and red, respectively.

[b]Macrocycles **3** and **5** do not populate state 2 in the MD simulations in $CDCl_3$.

[c]Determined at -20 °C

Abbreviations: br: broad, ol: overlap

Table S8. Comparison of $^{3}J_{H,H}$ coupling constants calculated for the state 1 and state 2 conformations of the MEC from protocols **MD:DFT** to those determined by NMR spectroscopy in DMSO-d_6 for compound **1**, **3**, and **5**[a]

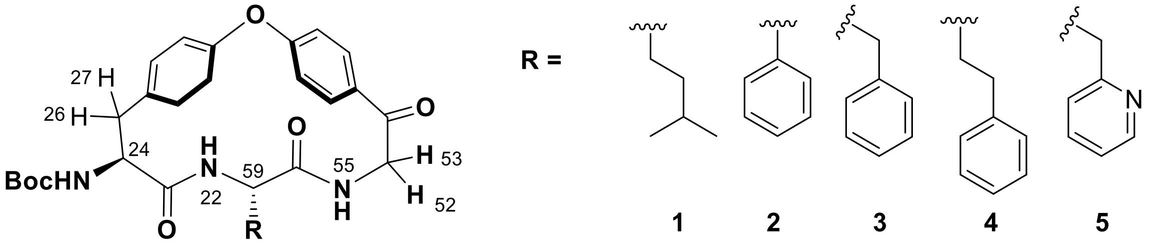

Cpd.	Protocol	J (Hz) NH-I 55-52	J (Hz) NH-I 55-53	J (Hz) NH-II 22-59	J (Hz) H-24 24-26	J (Hz) H-24 24-27
1	State 1	9.8 (175.1°)	2.2 (56.2°)	10.3 (175.8°)	11.2 (-178.7°)	3.6 (129.1°)
	State 2	0.6 (-108.8°)	6.1 (134.6°)	9.3 (178.1°)	10.2 (-177.0°)	3.6 (67.1 °)
	Exp.	8.5	br	7.8	12.0	2.7
3	State 1	9.8 (168.5°)	2.2 (49.7°)	10.2 (140.3°)	11.3 (178.1°)	3.6 (61.4°)
	State 2	1.4 (-64.8°)	9.5 (177.2°)	9.9 (168.7°)	11.2 (-179.8°)	3.7 (62.9°)
	Exp.	8.2	3.3	7.6	12.1	ol
5	State 1	9.9 (-171.4°)	1.0 (69.5°)	9.2 (165.0°)	11.3 (179.7°)	3.9 (62.1°)
	State 2	0.34 (-59.1°)	6.0 (-177.4°)	8.5 (171.3°)	10.9 (-175.6°)	5.0 (67.2°)
	Exp.	9.2	br	6.1	10.5	ol

[a]Cut-offs for the agreement between calculated and experimentally determined coupling constants: High: $\leq \pm 1$ Hz; Moderate: $\pm$ (1–2) Hz; Low: $\geq \pm 2$ Hz. Moderate and Low agreement has been marked in orange and red, respectively.
Abbreviations: br: broad, ol: overlap

Table S9. ^{1}H-^{1}H distances determined from NOESY spectra of compounds **1**, **3** and **5** recorded in $CDCl_3$ and DMSO-d_6[a]

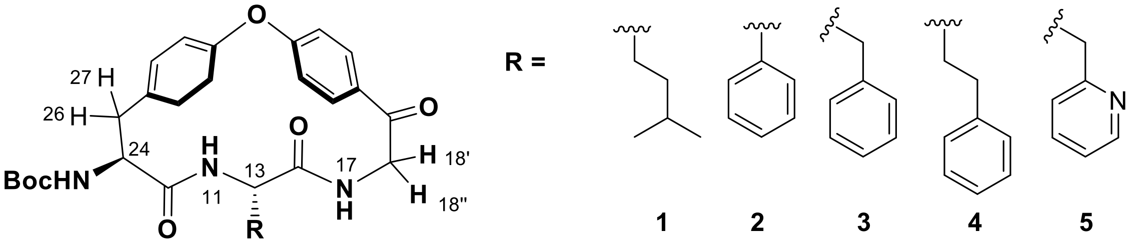

Compound No.	Solvent	13-17 (Å)	17-18''(Å)	17-18'(Å)	11-9 (Å)
**1**	$CDCl_3$	2.24	2.48	-	2.22
	DMSO-d_6	2.40	2.46	3.27	2.21
**3**	$CDCl_3$	2.13	2.41	-	2.12
	DMSO-d_6	2.22	2.49	2.94	2.04
**5**	$CDCl_3$	2.03	2.46	-	1.92
	DMSO-d_6	2.76	2.53	3.52	2.4

[a]The distance between H18' and H18'' (1.78 Å) was used as reference when calculating the distances

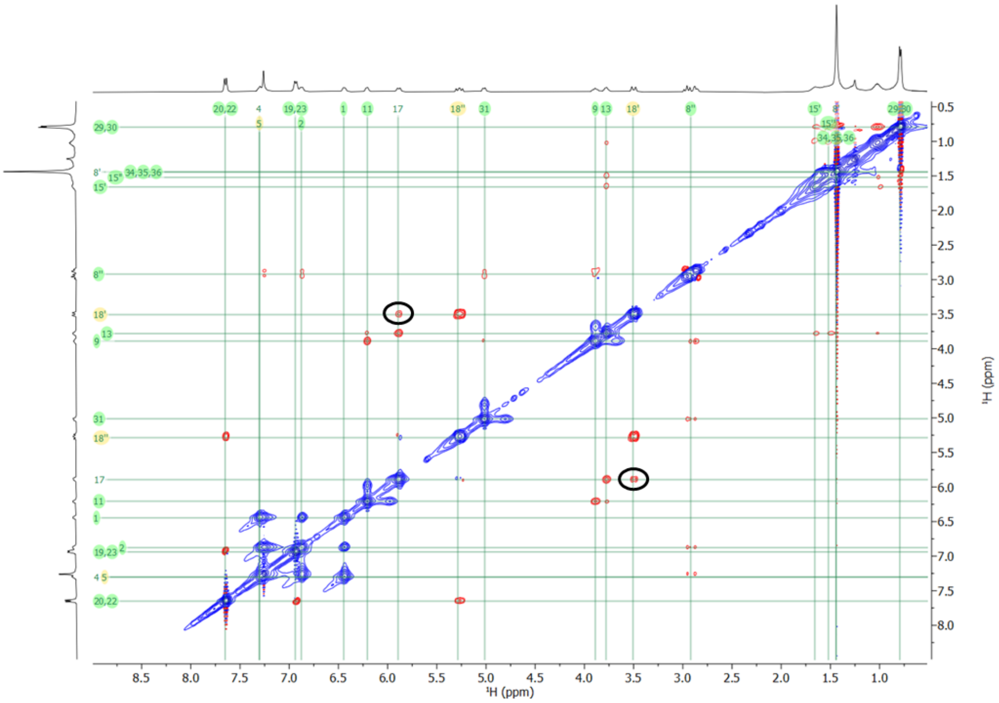

Figure S22. NOESY spectrum of compound **1** in $CDCl_3$ recorded with a mixing time of 300 ms at 400 MHz.

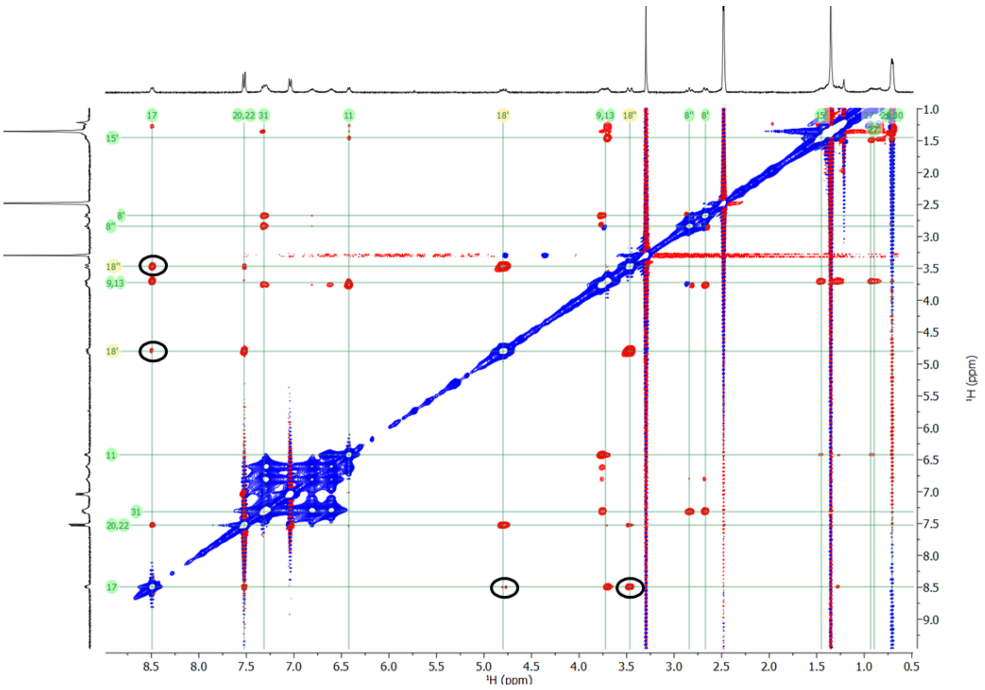

Figure S23. NOESY spectrum of compound **1** in DMSO-d_6 recorded with a mixing time of 300 ms at 400 MHz.

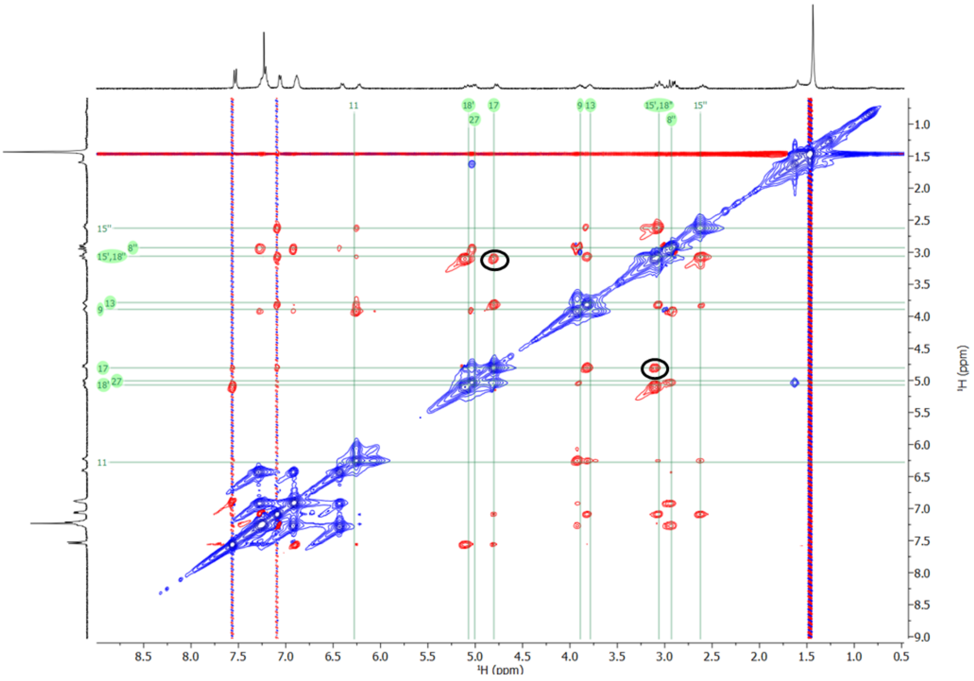

Figure S24. NOESY spectrum of compound **3** in $CDCl_3$ recorded with a mixing time of 300 ms at 400 MHz.

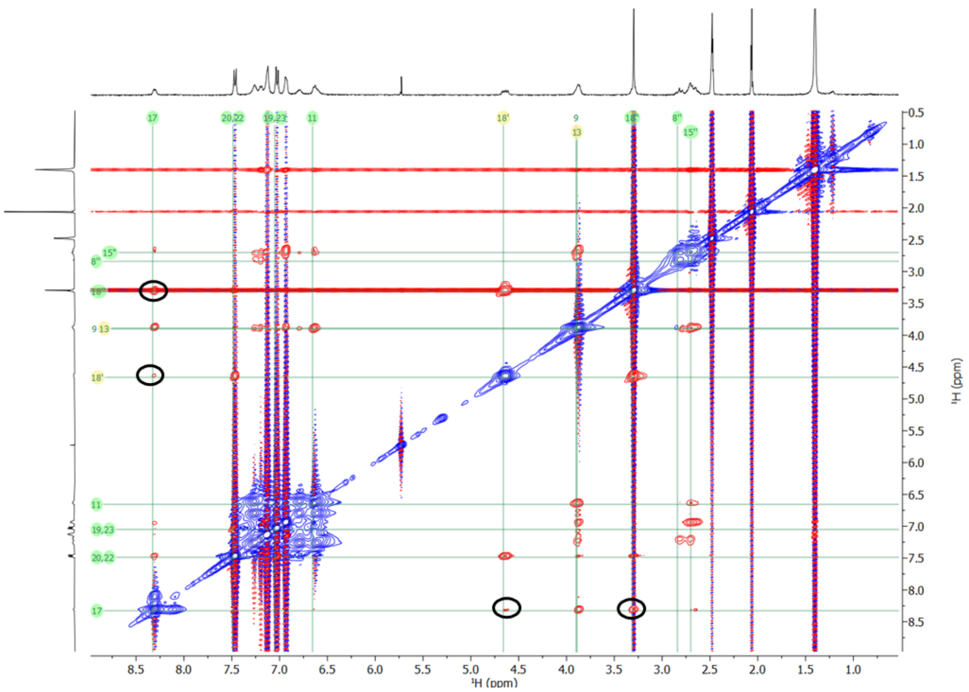

Figure S25. NOESY spectrum of compound **3** in DMSO-d_6 recorded with a mixing time of 300 ms at 400 MHz.

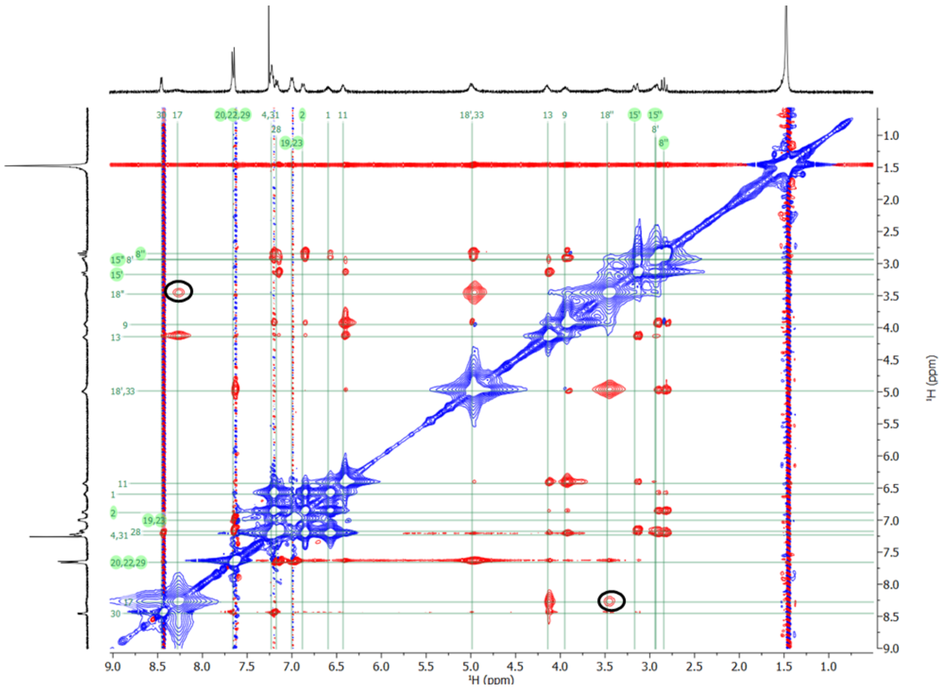

Figure S26. NOESY spectrum of compound **5** in $CDCl_3$ recorded with a mixing time of 300 ms at 400 MHz.

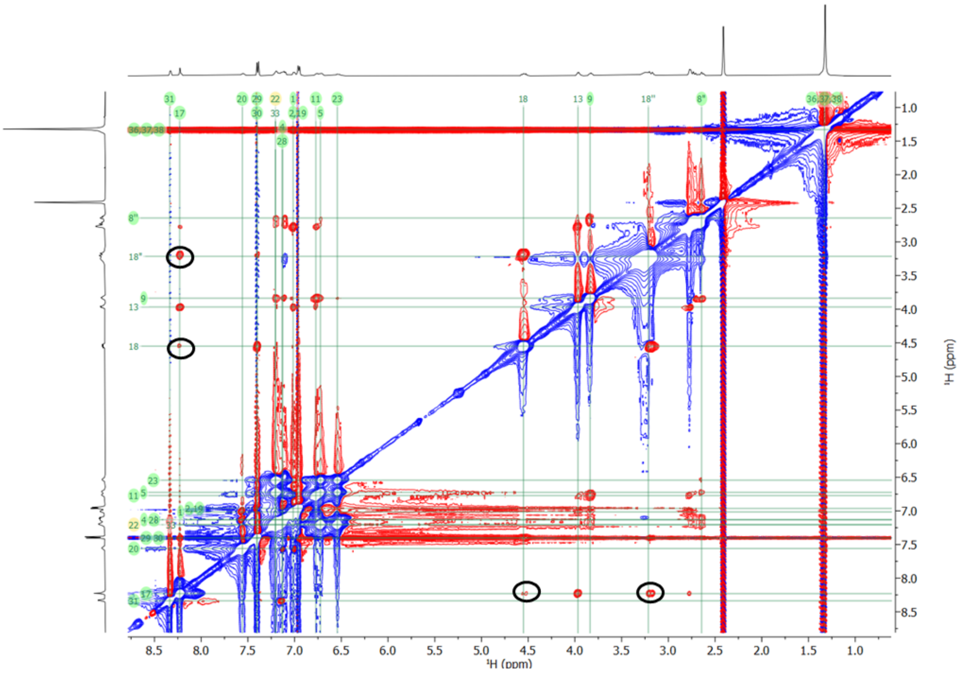

Figure S27. NOESY spectrum of compound **5** in DMSO-d_6 recorded with a mixing time of 300 ms at 400 MHz.

Synthesis of compound 5

General methods

Reagents and solvents were purchased from Sigma-Aldrich, Fluorochem, and VWR International. However, Boc-3-(2-pyridyl)-L-alanine (**S1**) was purchased from Acrotein ChemBio. 2-(4-Fluoro-3-nitrophenyl)-2-hydroxyethan-1-ammonium chloride **(S2)** and (*S*)-2-((*tert*-butoxycarbonyl)amino)-3-(4-((*tert*-butyldimethylsilyl)oxy)phenyl)propanoic acid **(S4)** were prepared using methods reported in literature. [1-3] Glassware used for carrying out experiments were oven dried. Solvents were evaporated on a Büchi rotavapor R-114. Hydrogenation was performed in a Parr hydrogenator (series 5100). Mainly LC-MS was used for monitoring reactions using an Agilent 1100 series HPLC having a C18 Atlantis T3 column (3.0 × 50 mm, 5 μm). Acetonitrile–water (flow rate 0.75 mL/min over 6 min) was used as mobile phase and a Waters micromass Z_Q (model code: MM1) mass spectrometer operating in electrospray ionization mode was used for detection of molecular ions. TLC were visualized by UV light (254 nm) and staining with phosphomolybdic acid in ethanol or ethanolic H_2SO_4 (5% v/v). Compounds were purified by flash column chromatography using silica gel (Matrex, 60 Å, 35–70 μm, Grace Amicon) and/or a Gilson HPLC equipped with a Kromasil C8 column (250 × 21.2 mm, 5 μm), a Gilson 322 pump, a UV/Visible-156 detector and a 202 collector. Acetonitrile-water gradients were used as eluents for HPLC purifications with a flow rate of 15 mL/min and detection at 214 or 254 nm.

^{1}H, ^{13}C, COSY, HSQC and HMBC NMR spectra for the synthesized compounds were recorded at 298 K on an Agilent Technologies 400 MR spectrometer at 400 MHz or 100 MHz. The residual peak of the respective solvent was used as internal standard [$CDCl_3$ ($CHCl_3$ δ_H 7.26 ppm, $CDCl_3$ δ_C 77.0 ppm) or CD_3SOCD_3 (CD_2HSOCD_3 δ_H 2.50 ppm, CD_3SOCD_3 δ_C 39.5 ppm)]. COSY, HSQC, and HMBC spectra were used for assigning the protons of the macrocycles. Since compounds **S3**, **S5**, and **S6** were prepared as mixtures of diastereomers leading to highly complex spectra the ^{1}H and ^{13}C NMR were not assigned, but only the observed peaks are reported. HRMS for all new synthesized compounds used in this manuscript were recorded on LCT Premier mass spectrometer connected to a Waters acquity UPLC I-class operating in electrospray ionization (ESI) and APCI mode using acetonitrile:water as mobile phase (1:1, with a flow rate of 0.25 mL/min).

Synthetic scheme for compound 5

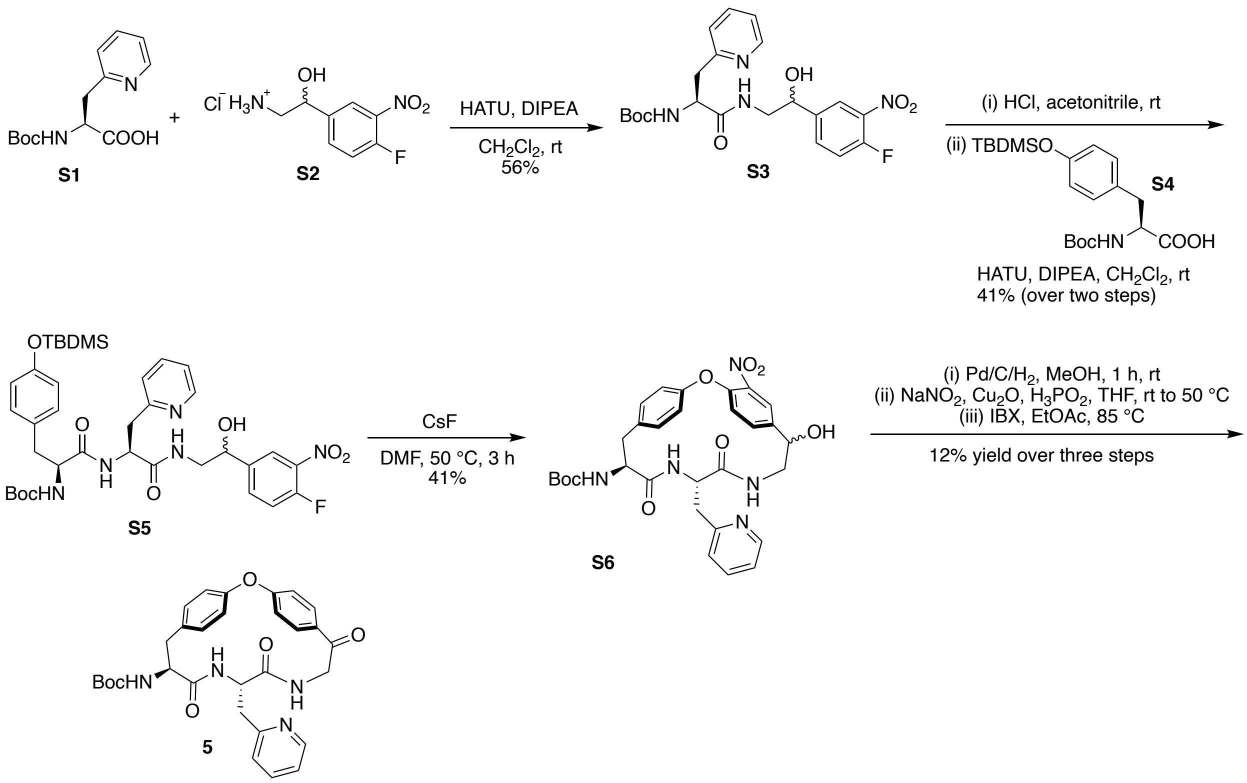

Scheme 1. Synthesis of compound **5**

Experimental procedures

***tert*-butyl ((2*S*)-1-((2-(4-fluoro-3-nitrophenyl)-2-hydroxyethyl)amino)-1-oxo-3-(pyridin-2-yl)propan-2-yl)carbamate (S3)**

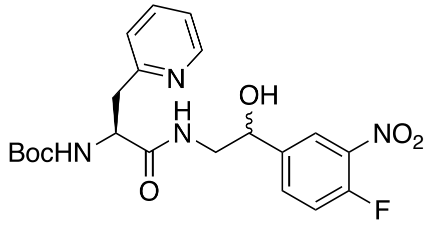

Boc-3-(2-pyridyl)-L-alanine (**S1**) (2.18 g, 8.20 mmol) and HATU (3.68 g, 9.70 mmol) were dissolved in DCM (25 mL). After stirring for 10 min at room temperature, DIPEA (2.86 mL, 16.4 mmol) and **S2**[1] (1.50 g, 6.33 mmol) were added followed by addition of another portion of DIPEA (2.86 mL, 16.4 mmol). TLC showed complete conversion after stirring at room temperature for 40 min. The reaction mixture was concentrated under reduced pressure and purified on a silica gel column using 80% EtOAc in *n*-hexane to 10% MeOH in EtOAc as mobile phase to give **S3** (1.58 g, 3.54 mmol, 56%) as a blackish viscous liquid which was used directly in the next step. A small

portion of this material was further purified on reverse phase HPLC using a gradient from 10% to 75% acetonitrile in water to provide **S3** as a white powder for characterization.

HRMS (ESI) m/z calcd for $C_{21}H_{26}N_4O_6F$ $[M + H]^+$ 449.1836, found 449.1842.

^{1}H NMR (400 MHz, $CDCl_3$) δ 8.43–8.39 (m, 2H), 8.11–8.05 (m, 2H), 7.70–7.60 (m, 4H), 7.45–7.38 (m, 1H), 7.25–7.19 (m, 7H), 6.17–6.15 (m, 1H), 6.01–5.99 (m, 1H), 4.98–4.87 (m, 2H), 4.62–4.55 (m, 2H), 3.79–3.51 (m, 3H), 3.42–3.04 (m, 8H), 1.41 (2s, 18H).

^{13}C NMR (101 MHz, $CDCl_3$) δ 172.2, 157.2, 156.7, 155.9, 155.9, 153.3, 153.3, 148.2, 148.1, 139.0, 139.0, 138.9, 138.9, 137.6, 137.5, 137.2, 137.1, 132.9, 132.8, 132.7, 125.0, 124.7, 123.4, 123.4, 123.4, 123.3, 122.3, 122.2, 118.4, 118.4, 118.2, 118.2, 80.2, 71.4, 70.2, 54.0, 53.7, 47.8, 47.5, 39.9, 39.4, 28.3, 28.2.

***tert*-butyl ((2*S*)-3-(4-((*tert*-butyldimethylsilyl)oxy)phenyl)-1-(((2*S*)-1-((2-(4-fluoro-3-nitrophenyl)-2-hydroxyethyl)amino)-1-oxo-3-(pyridin-2-yl)propan-2-yl)amino)-1-oxopropan-2-yl)carbamate (S5)**

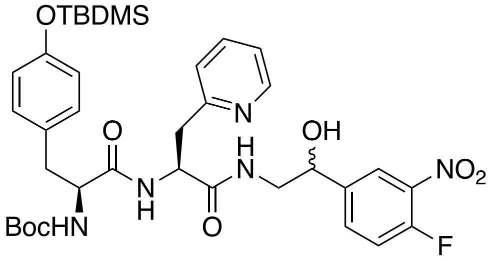

Compound **S3** (1.26 g, 2.80 mmol) was dissolved in acetonitrile (20 mL), then an aqueous conc. HCl solution (4.00 mL) was added at room temperature and stirring was continued for another 30 min. The reaction mixture was concentrated under reduced pressure, co-evaporated with toluene (10 mL), and further dried under high vacuum for 1 h. Then compound **S4**[2, 3] (1.44 g, 3.64 mmol), HATU (1.26 g, 3.31 mmol) was dissolven in DIPEA (1.15 mL, 6.64 mmol) in DCM (25 mL), followed by addition of another portion of DIPEA (1.15 mL, 6.64 mmol). LC-MS showed complete conversion after stirring at room temperature for 40 min. The reaction mixture was concentrated under reduced pressure and purified on a silica gel column using EtOAc to 10% MeOH in EtOAc as mobile phase to give **S5** (833 mg, 1.14 mmol, 41%) as a blackish viscous liquid which was used directly for the next step. A small portion of this material was further purified on reverse phase HPLC using a gradient from 40% to 95% acetonitrile in water to provide **S5** as a white powder for characterization.

HRMS (ESI) m/z calcd for $C_{36}H_{49}N_5O_8FSi$ $[M + H]^+$ 726.3334, found 726.3336.

^1H NMR (400 MHz, $CDCl_3$) δ 8.45–8.38 (m, 2H), 8.07–8.04 (m, 1H), 7.87–7.77 (m, 1H), 7.65–7.58 (m, 1H), 7.50–7.28 (m, 2H), 7.25–7.16 (m, 1H), 7.13–7.01 (m, 3H), 6.82–6.71 (m, 2H), 5.03–4.93 (m, 1H), 4.88–4.80 (m, 1H), 4.77–4.73 (m, 1H), 4.28–4.15 (m, 1H), 3.47–3.42 (m, 2H), 3.31–2.82 (m, 6H), 1.38–1.37 (m, 9H), 0.95–0.93 (m, 9H), 0.17–0.12 (m, 6H).

^{13}C NMR (101 MHz, $CDCl_3$) δ 171.7, 171.1, 156.4, 154.8, 139.0, 137.1, 132.9, 130.1, 128.3, 123.5, 120.5, 120.3, 118.2, 118.0, 81.1, 71.7, 71.1, 56.9, 52.8, 52.6, 48.0, 47.8, 36.7, 36.3, 28.2, 25.6, 18.1, -4.4.

***tert*-butyl ((8*S*,11*S*)-4-hydroxy-3^2-nitro-7,10-dioxo-8-(pyridin-2-ylmethyl)-2-oxa-6,9-diaza-1,3(1,4)-dibenzenacyclododecaphane-11-yl)carbamate (S6)**

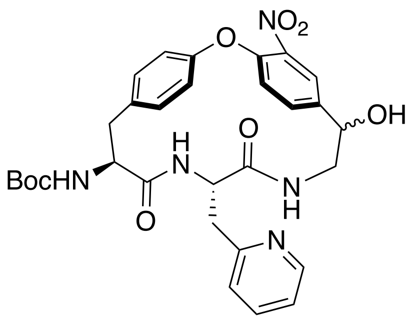

Compound **S5** (725 mg, 1.00 mmol) was dissolved in DMF (100 mL) and CsF (3.03 g, 20.0 mmol) was added to it portion wise over 45 min at 50 ºC. LC-MS showed good conversion after 3 h. DMF was evaporated under reduced pressure at 70 ºC, the residue was purified on a silica gel column using 10% to 40% MeOH in EtOAc as mobile phase to give **S6** (242 mg, 0.410 mmol, 41%) as a blackish viscous liquid. A part of this was further purified on reverse phase HPLC using a gradient from 10% to 75% acetonitrile in water to provide **S6** as a colorless powder for characterization.

HRMS (ESI) m/z calcd for $C_{30}H_{32}N_5O_8$ $[M - H]^-$ 590.2251, found 590.2255.

^1H NMR (400 MHz, DMSO-d_6) δ 8.48–8.38 (bs, 1H), 7.98–7.96 (m, 1H), 7.83–7.79 (m, 1H), 7.70–7.58 (m, 1H), 7.51–7.45 (m, 1H), 7.42–6.80 (m, 9H), 6.54–6.41 (m, 1H), 5.82–5.71 (m, 1H), 4.95 (s, 1H), 4.56–4.49 (m, 1H), 4.16–4.03 (m, 2H), 3.79–3.66 (m, 2H), 3.04–2.78 (m, 4H), 2.72–2.56 (m, 2H), 1.37 (s, 9H).

^{13}C NMR (101 MHz, DMSO-d_6) δ 169.9, 159.2, 159.0, 155.3, 152.8, 152.4, 142.8, 140.6, 140.1, 134.5, 134.5, 133.3, 132.9, 131.3, 124.7, 122.2, 121.8, 78.6, 70.4, 51.8, 51.5, 49.0, 37.0, 28.6.

***tert*-butyl ((8*S*,11*S*)-4,7,10-trioxo-8-(pyridin-2-ylmethyl)-2-oxa-6,9-diaza-1,3(1,4)-dibenzenacyclododecaphane-11-yl)carbamate (5)**

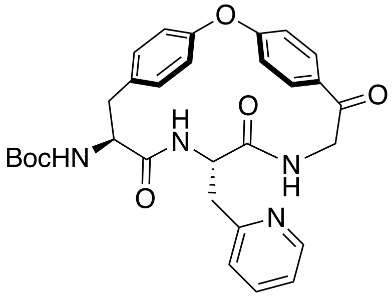

Compound **S6** (240 mg, 0.400 mmol) was added to Pd/C (10%, 240 mg, 0.22 mmol Pd) followed by addition of MeOH (8 mL) under an argon atmosphere. The atmosphere was exchanged to 1 atm. H_2 via purging the system three times with hydrogen in a Parr hydrogenator. LC-MS analysis showed complete conversion after stirring at room temperature for 1 h. The reaction mixture was filtered through a pad of celite, concentrated, and dried under reduced pressure. Then THF (8 mL) was added followed by addition of 50% aqueous hypophosphorus acid (374 µL, 6.9 mmol), Cu_2O (2.3 mg, 16.1 µmol), and a sodium nitrite (43.7 mg, 0.63 mmol) solution in water (2 mL) and stirred at 50 ºC. LC-MS showed complete conversion with formation of side products within 10 min. The reaction was allowed to cool to room temperature and ethyl acetate (100 mL) was added, then the organic phase was washed with brine (2 × 50 mL), dried over anhydrous Na_2SO_4, filtered, and concentrated under reduced pressure. The crude was dissolved in ethyl acetate (10 mL) and DMSO (2 mL), and IBX (45 wt.%, 555 g, 0.89 mmol) was added portion wise over 30 min with stirring at 85 ºC and monitoring by LC-MS. After 30 min, the reaction mixture was allowed to cool to room temperature, centrifuged (to remove undissolved IBX), concentrated under reduced pressure to 5 mL and centrifuged (to remove undissolved IBX) again, concentrated and purified on reverse phase HPLC using a gradient from 10% to 70% acetonitrile in water to give **5** (32 mg, 0.06 mmol, 12%) as a colorless powder.

HRMS (ESI) m/z calcd for $C_{30}H_{31}N_4O_6$ $[M-H]^-$ 543.2244, found 543.2238.

^{1}H NMR (400 MHz, $CDCl_3$) δ 8.45 (d, *J* = 5.1 Hz, 1H), 8.16 (s, 1H), 7.70 (t, *J* = 7.7 Hz, 1H), 7.64 (d, *J* = 8.4 Hz, 2H), 7.24 (m, 4H), 7.07–6.81 (m, 3H), 6.58 (s, 1H), 6.43 (s, 1H), 5.00 (m, 2H), 4.22–4.06 (m, 1H), 3.94 (s, 1H), 3.48 (s, 1H), 3.25–2.76 (m, 4H), 1.47 (s, 9H).

^{13}C NMR (101 MHz, $CDCl_3$) δ 199.5, 170.0, 169.1, 164.7, 160.1, 156.8, 154.9, 147.1, 138.5, 132.7, 131.4, 130.9, 130.2, 129.6, 124.9, 123.1, 122.7, 122.4, 121.5, 80.2, 57.8, 51.8, 47.8, 39.8, 38.1, 28.3.

NMR spectra of compounds S3, S5, S6 and 5

***tert*-butyl ((2*S*)-1-((2-(4-fluoro-3-nitrophenyl)-2-hydroxyethyl)amino)-1-oxo-3-(pyridin-2-yl)propan-2-yl)carbamate** (S3)

^{1}H NMR in $CDCl_3$:

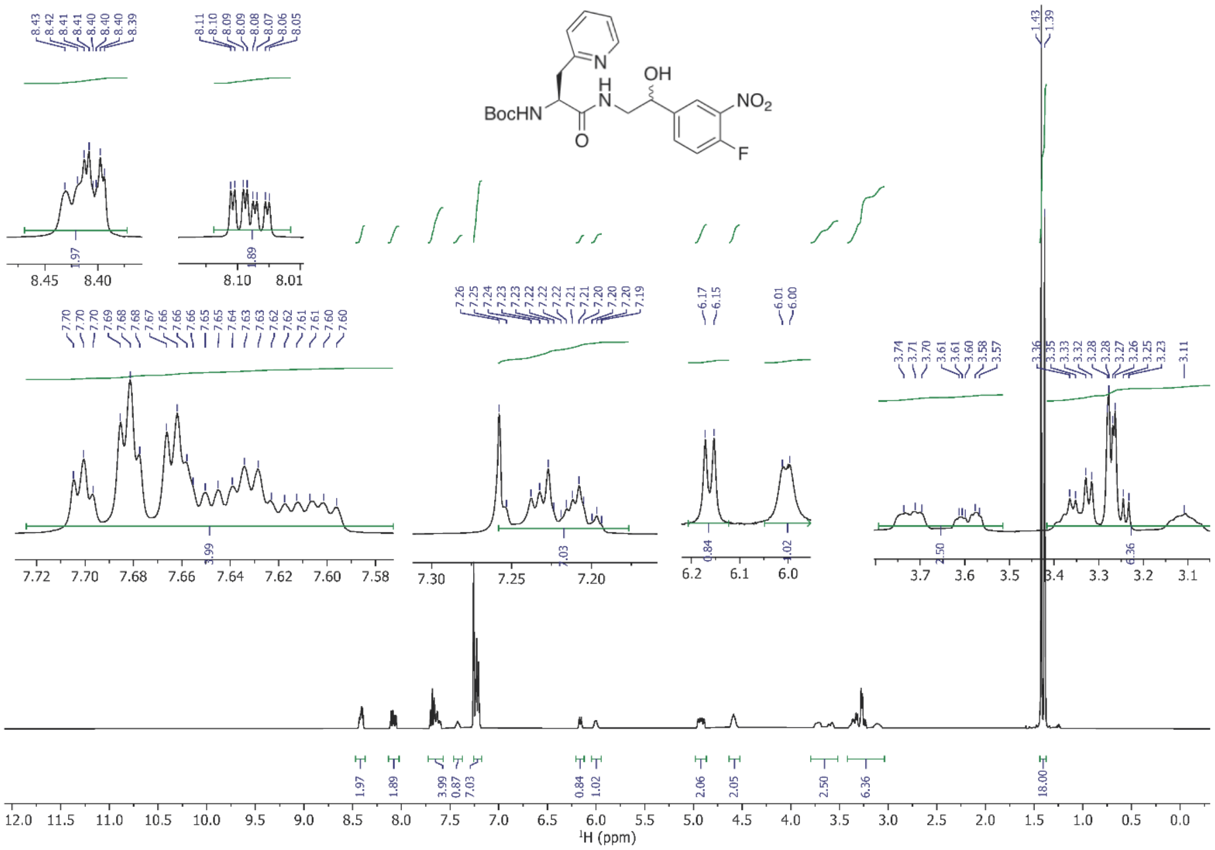

^{13}C NMR in $CDCl_3$:

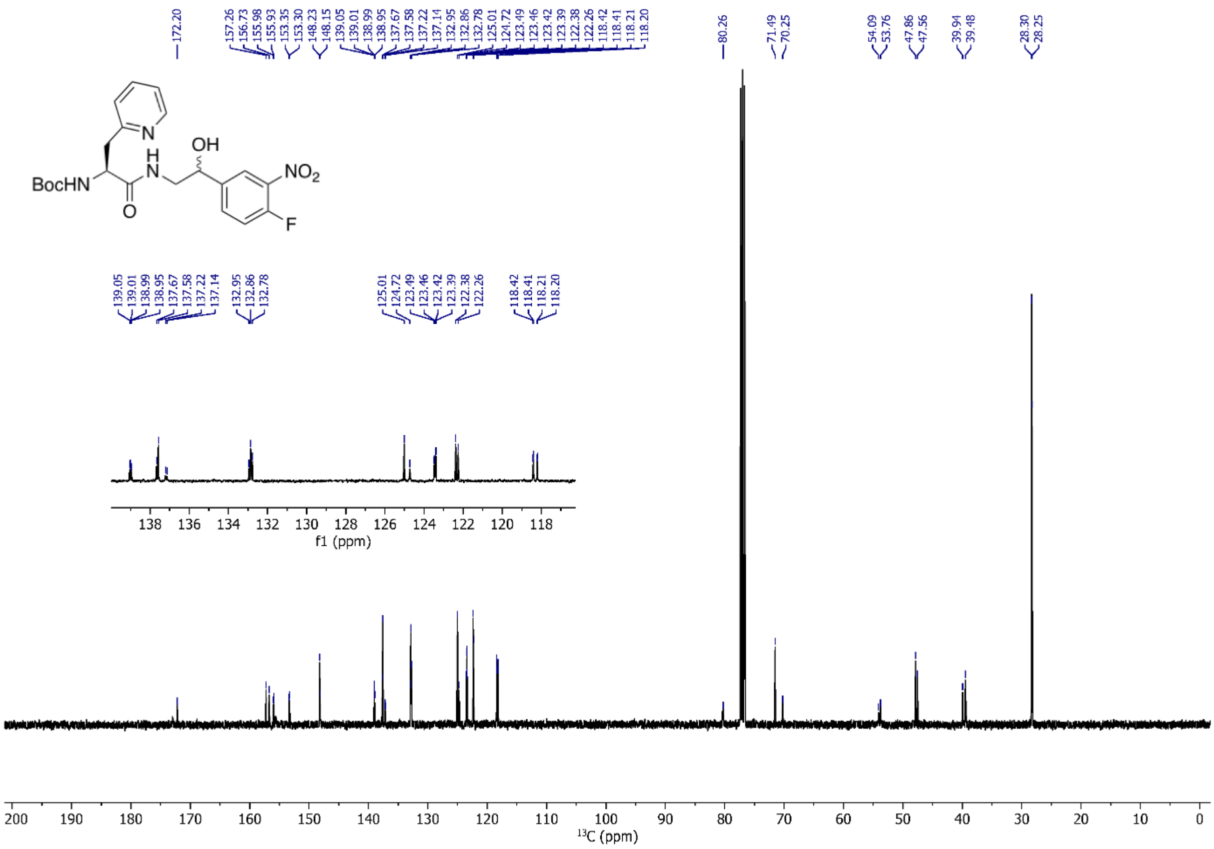

***tert*-butyl ((2*S*)-3-(4-((*tert*-butyldimethylsilyl)oxy)phenyl)-1-(((2*S*)-1-((2-(4-fluoro-3-nitrophenyl)-2-hydroxyethyl)amino)-1-oxo-3-(pyridin-2-yl)propan-2-yl)amino)-1-oxopropan-2-yl)carbamate** (S5)

^{1}H NMR in $CDCl_3$:

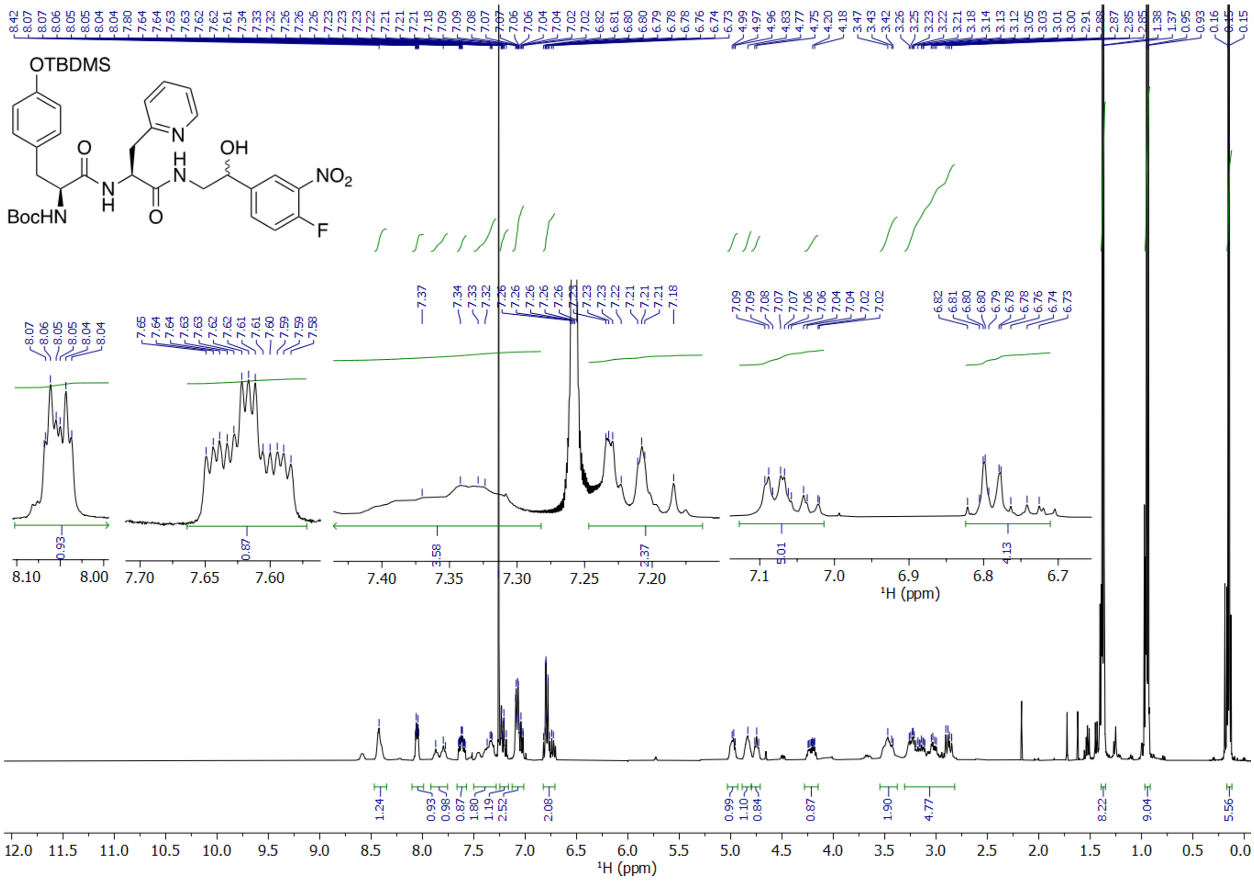

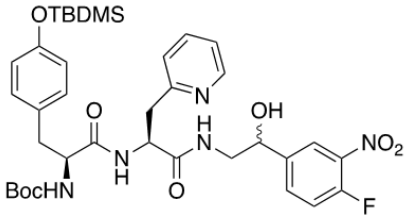

OTBDMS
O
N
H
OH
NO2
BocHN
O
F

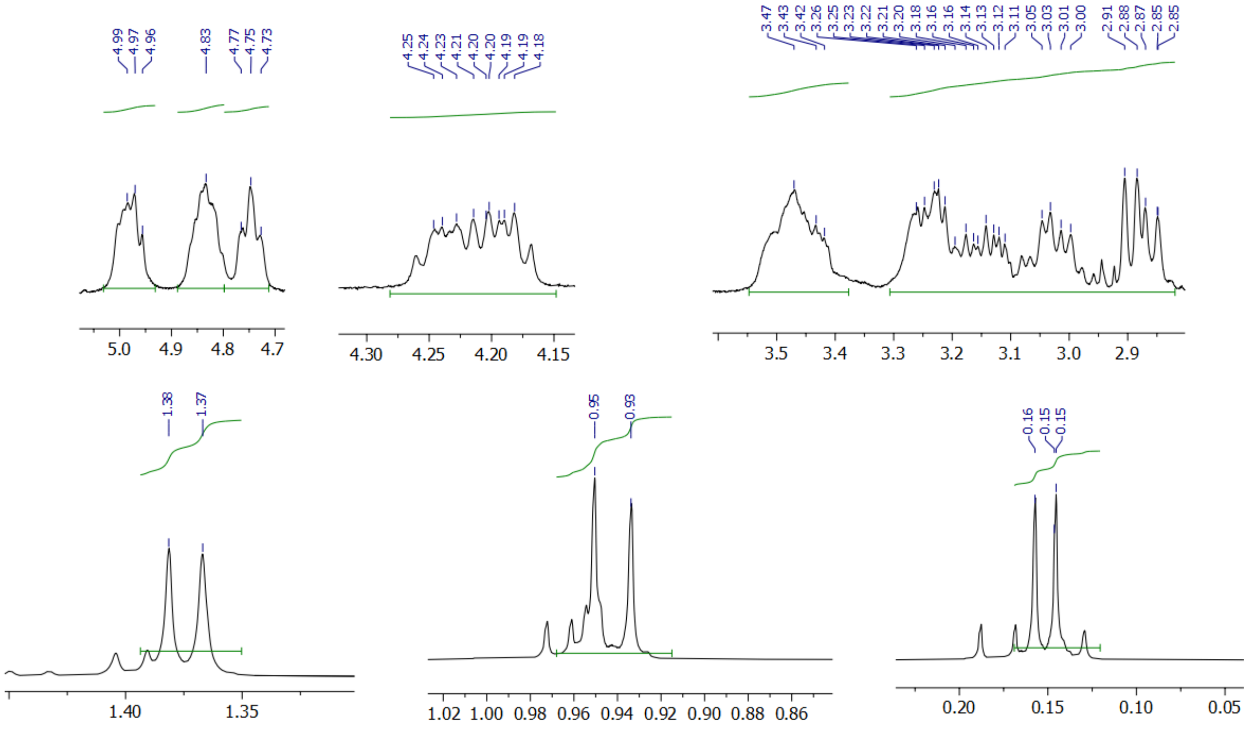

^{13}C NMR in $CDCl_3$:

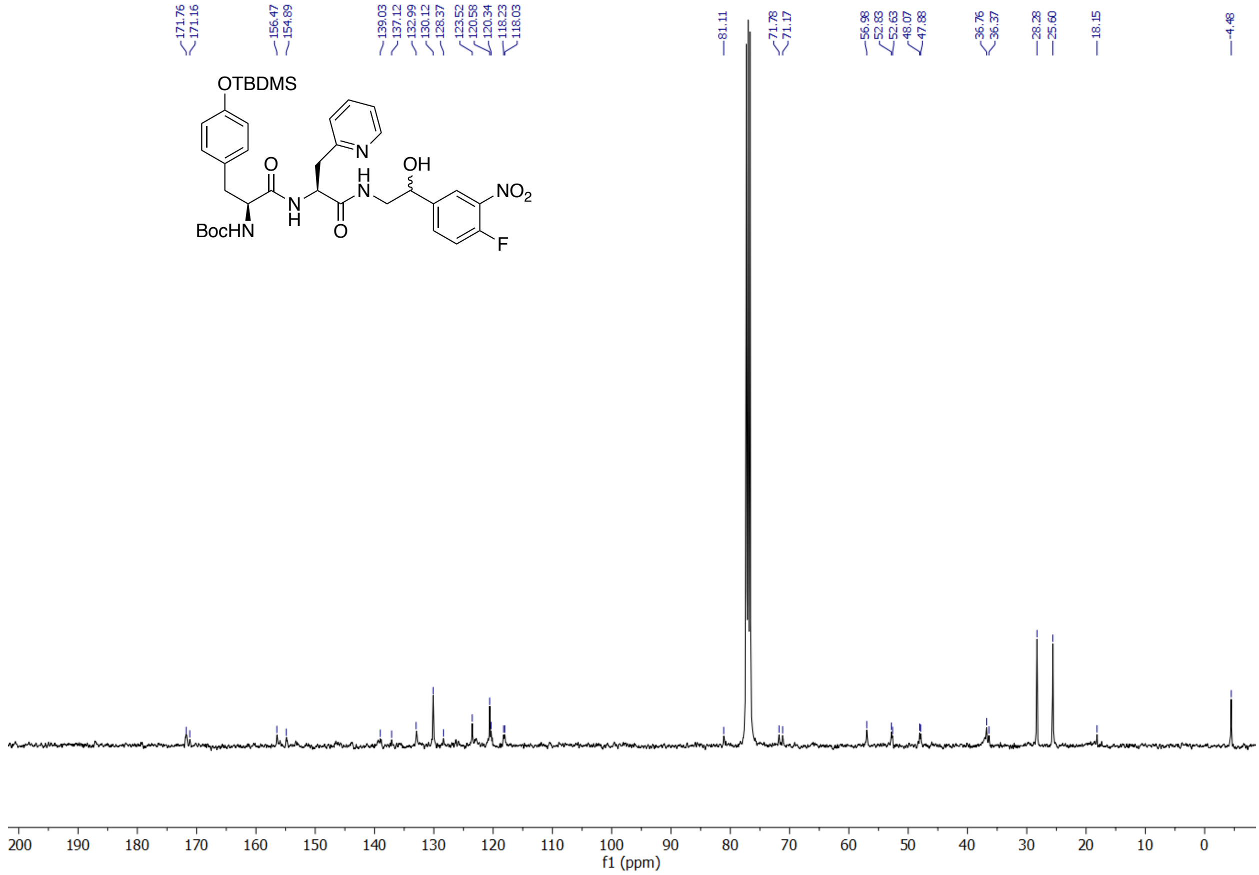

***tert*-butyl ((8*S*,11*S*)-4-hydroxy-3²-nitro-7,10-dioxo-8-(pyridin-2-ylmethyl)-2-oxa-6,9-diaza-1,3(1,4)-dibenzenacyclododecaphane-11-yl)carbamate** (S6)

¹H NMR in DMSO-d₆:

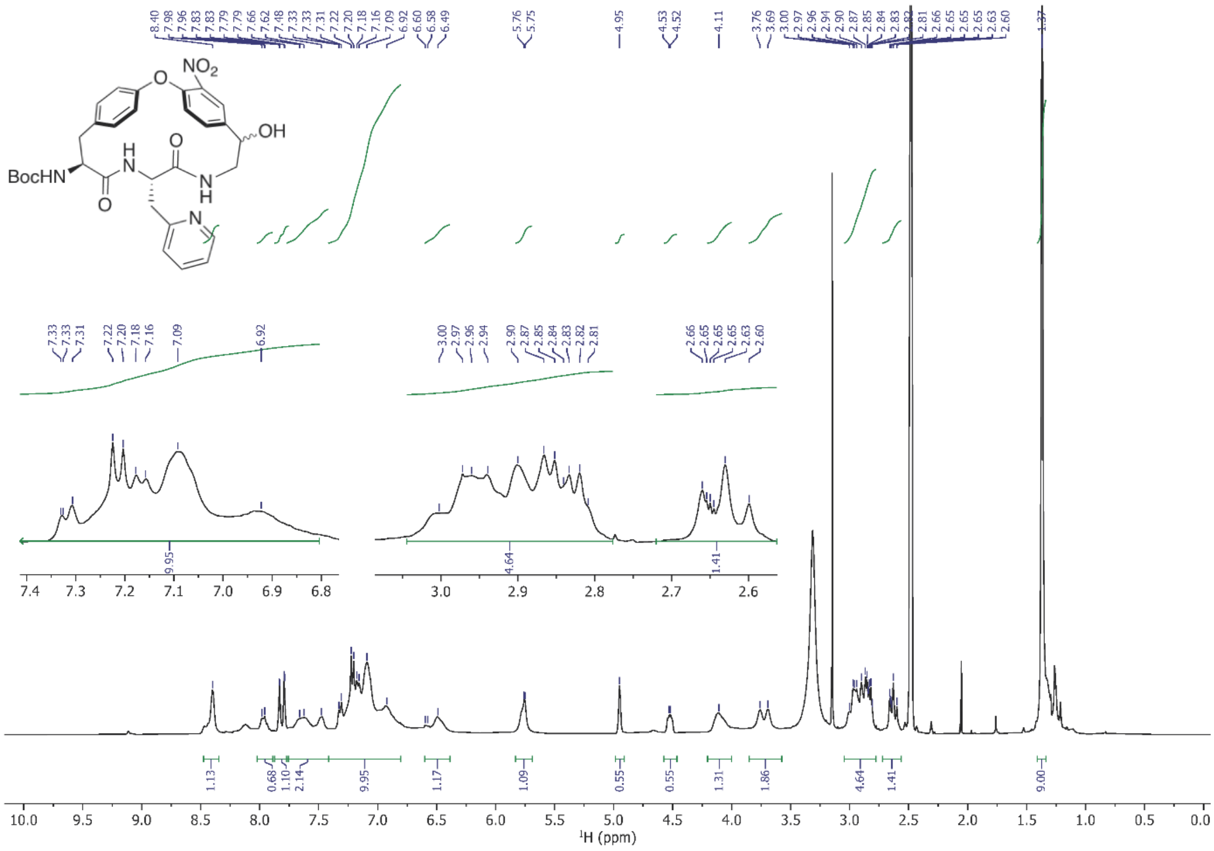

^{13}C NMR in DMSO-d$_6$:

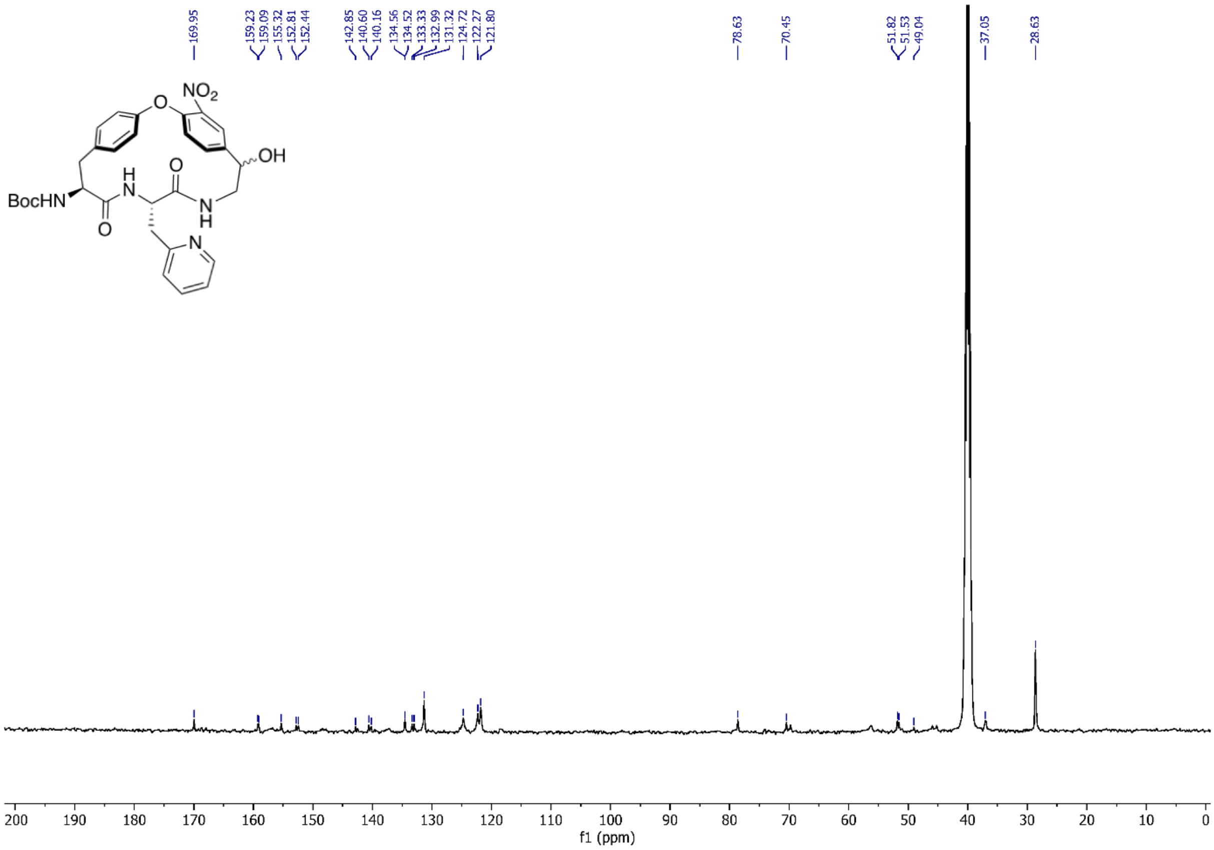

***tert*-butyl ((8*S*,11*S*)-4,7,10-trioxo-8-(pyridin-2-ylmethyl)-2-oxa-6,9-diaza-1,3(1,4)-dibenzenacyclododecaphane-11-yl)carbamate (5)**

^{1}H NMR in $CDCl_3$:

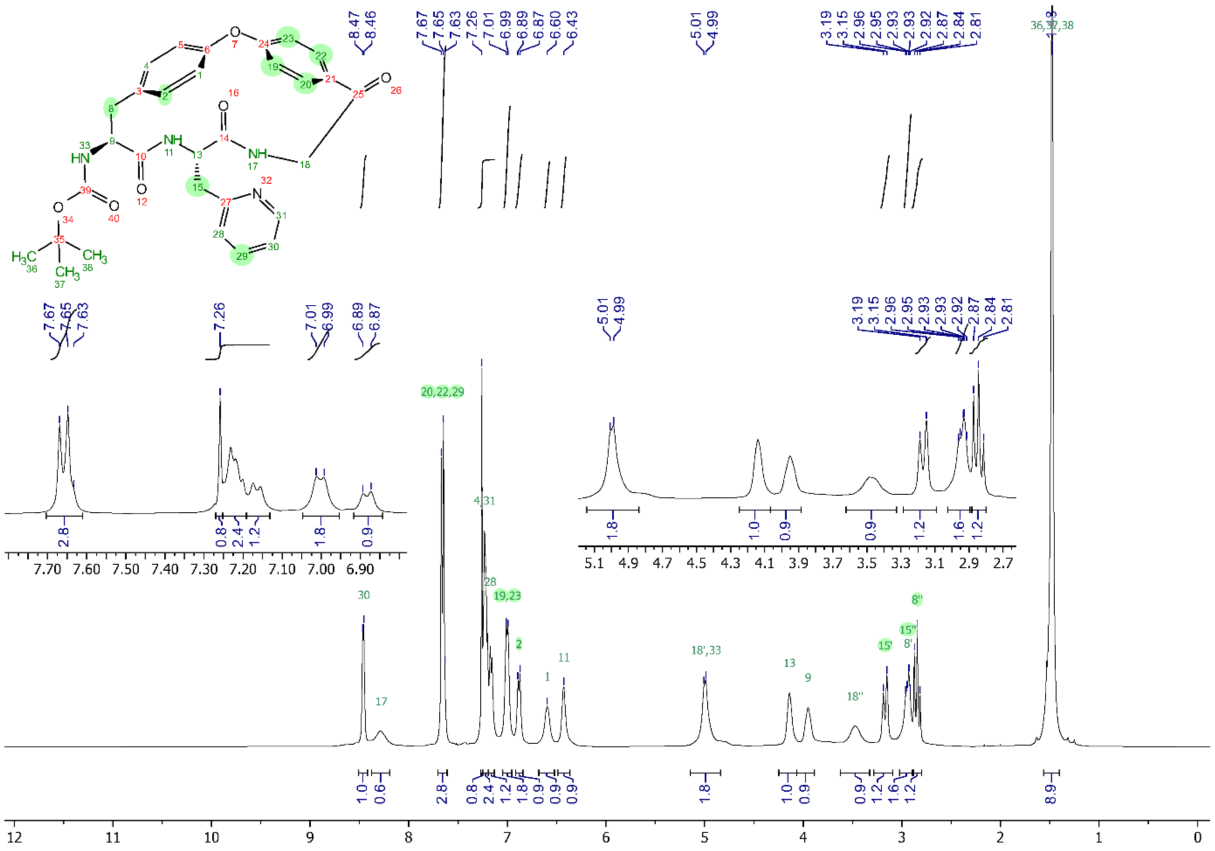

^{13}C NMR in $CDCl_3$:

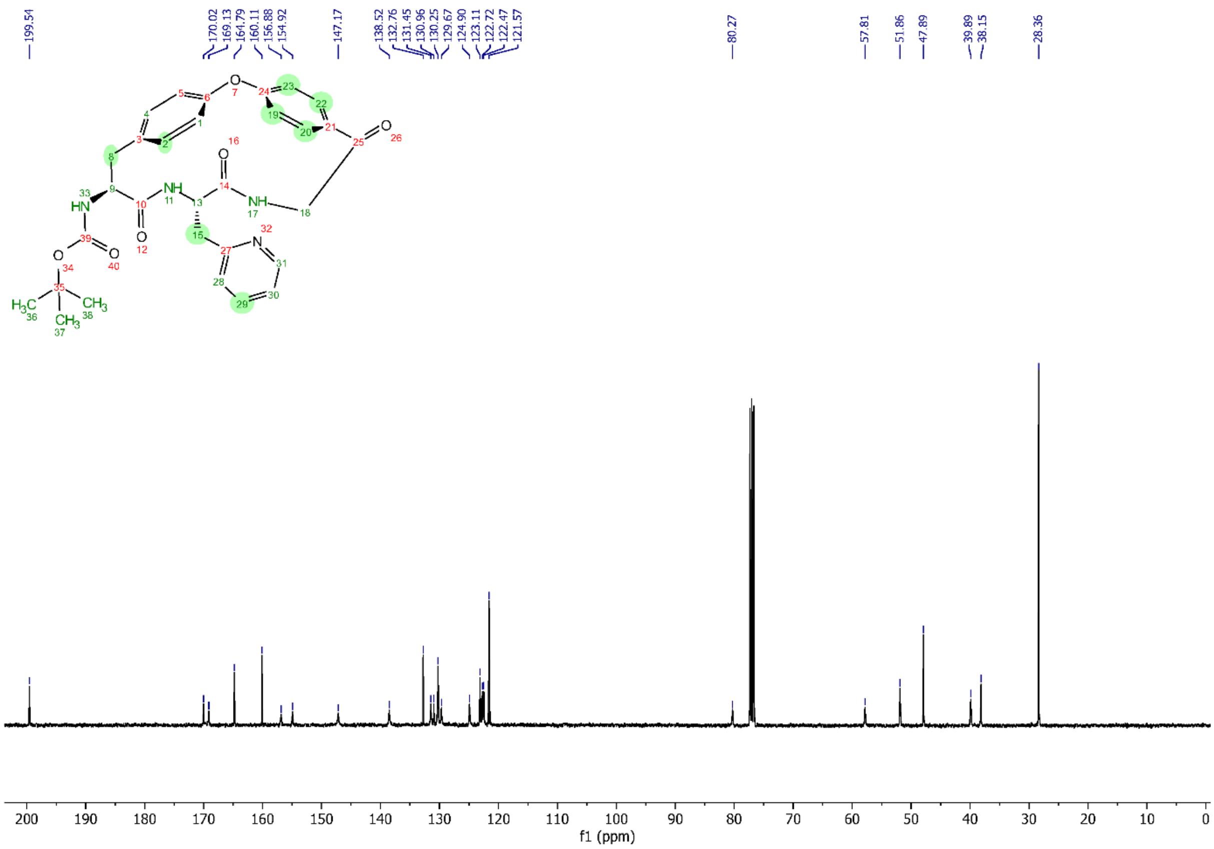

COSY in $CDCl_3$:

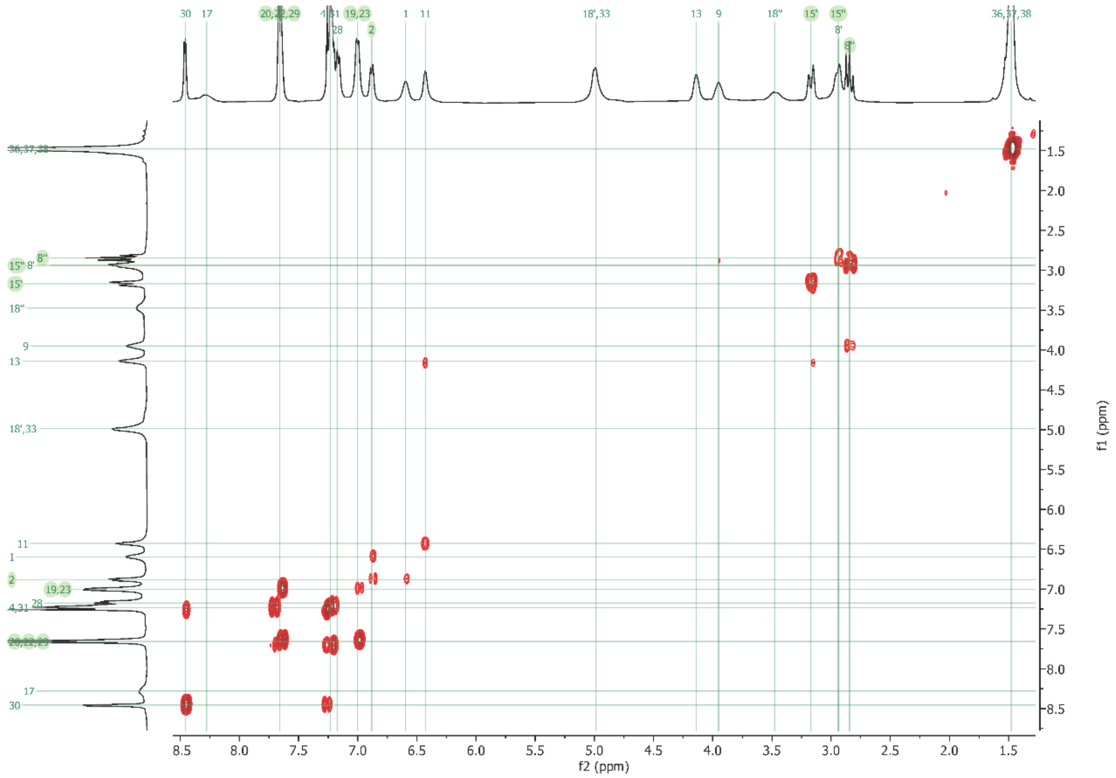

HSQC in $CDCl_3$:

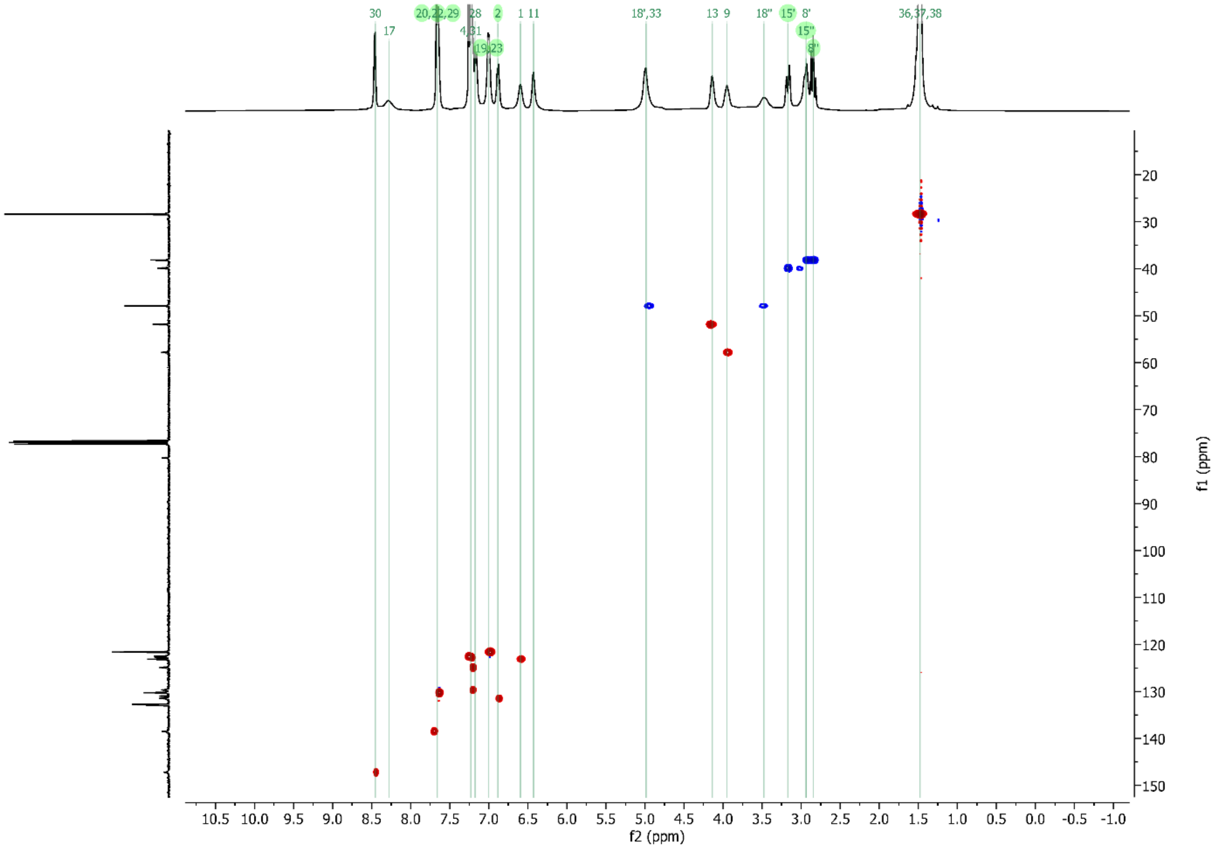

References

1. Laı¨b, T.; Ouazzani, J.; Zhu, J., Horse liver esterase catalyzed enantioselective hydrolysis of N,O-diacetyl-2-amino-1-arylethanol. *Tetrahedron: Asymmetry* **1998,** *9*, 169-178.
2. Bruckner, S.; Bilitewski, U.; Schobert, R., Synthesis and Antibacterial Activity of Four Stereoisomers of the Spider-Pathogenic Fungus Metabolite Torrubiellone D. *Org. Lett.* **2016,** *18*, 1136-1139.
3. Hansen, D. W.; Pilipauskas, D., Chemoselective N-ethylation of Boc amino acids without racemization. *J. Org. Chem* **1985,** *50*, 945-950.
